# Evolution of Mitochondria Reconstructed from the Energy Metabolism of Living Bacteria

**DOI:** 10.1371/journal.pone.0096566

**Published:** 2014-05-07

**Authors:** Mauro Degli Esposti, Bessem Chouaia, Francesco Comandatore, Elena Crotti, Davide Sassera, Patricia Marie-Jeanne Lievens, Daniele Daffonchio, Claudio Bandi

**Affiliations:** 1 Italian Institute of Technology, Genoa, Italy; 2 Department of Food, Environmental and Evolutionary Sciences, University of Milan, Milan, Italy; 3 Dipartimento di Scienze Veterinarie e Sanità Pubblica, University of Milan, Milan, Italy; Oregon Health & Science University, United States of America

## Abstract

The ancestors of mitochondria, or proto-mitochondria, played a crucial role in the evolution of eukaryotic cells and derived from symbiotic α-proteobacteria which merged with other microorganisms - the basis of the widely accepted endosymbiotic theory. However, the identity and relatives of proto-mitochondria remain elusive. Here we show that methylotrophic α-proteobacteria could be the closest living models for mitochondrial ancestors. We reached this conclusion after reconstructing the possible evolutionary pathways of the bioenergy systems of proto-mitochondria with a genomic survey of extant α-proteobacteria. Results obtained with complementary molecular and genetic analyses of diverse bioenergetic proteins converge in indicating the pathway stemming from methylotrophic bacteria as the most probable route of mitochondrial evolution. Contrary to other α-proteobacteria, methylotrophs show transition forms for the bioenergetic systems analysed. Our approach of focusing on these bioenergetic systems overcomes the phylogenetic impasse that has previously complicated the search for mitochondrial ancestors. Moreover, our results provide a new perspective for experimentally re-evolving mitochondria from extant bacteria and in the future produce synthetic mitochondria.

## Introduction

A major concept in biology is that the evolution of eukaryotic cell followed a symbiotic event between diverse microorganisms [1]–[4]. Mitochondria are the remnants of one of the original partners of this symbiotic event and in all likelyhood are related to extant α-proteobacteria [1]–[4]. However, the identity of the proto-mitochondrion remains elusive [1]. Phylogenetic studies suggested a relationship with endocellular parasites of the Rickettsiales order [4], [5], which has not been confirmed in subsequent reports [6]–[8]. Indeed, there appears to be a “phylogenetic impasse” in the identification of the partners that merged into the ancestral symbiotic progenitor of current eukaryotic cells [9], partly due to the problem of long branch attraction blurring the true geneology of living organisms and the fast evolution of mitochondrial DNA [1], [10].

The diverse metabolic processes carried out by living bacteria provide complementrary approaches to reconstruct key characteristics of the mitochondrial ancestors [11]. Although widely accepted, the reconstruction of proto-mitochondrial metabolism [12] has been partially contradicted by recent evidence suggesting that proto-mitochondria could be related to facultatively anaerobic generalists such as *Rhodobacter* [6]–[8], [10] - which are also capable of anoxygenic photosynthesis, an autotrophic function that must have been lost early along the evolution of mitochondria. Conversely, this evidence has recently been challenged by controversial reports that aerobic marine organisms such as *Pelagibacter ubique* may be the closest living relatives of mitochondria [13]–[15]. Other bacterial genera have also been considered to be phylogenetically related, or to display some analogies to the proto-mitochondrion: *Rhodospirillum* on the basis of extensive protein analysis [16]; *Paracoccus* for bioenergy considerations [1], and more recently following the evolution of complex I [17]; *Caulobacter*, on the basis of the sequence similarity of its homologues to the mitochondrial transport protein Tim44 [18]; *Micavibrio*, for its predatory ectoparasite character [19]; the Rhizobiales, *Ochrobactrum* and *Rhodopseudomonas*, for having many proteins in sister position to their mitochondrial homologues [6]–[8], [20]; and finally *Midichloria*, which appears to be the sole representative of the Rickettsiales retaining ancestral features typical of free-living bacteria [21]. The wide diversity of the proposed bacterial ancestors of mitochondria arises from the different approaches of molecular evolution that have been used and the inherent limits of such approaches [1]–[4].

**Figure 1:**
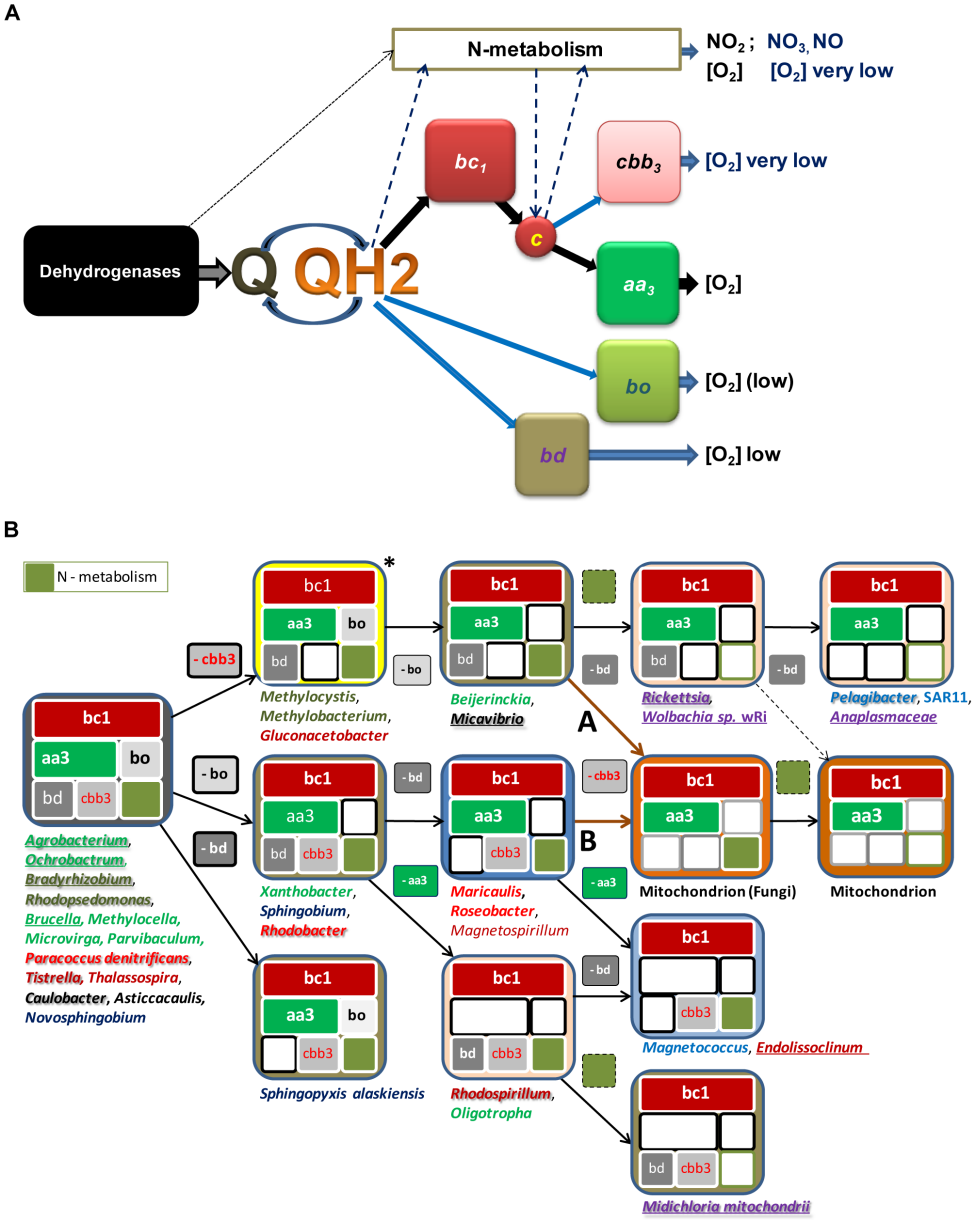
Bioenergetic systems of bacteria and mitochondria. **A -Terminal respiratory chain of bacteria.** 11. Various bioenergetic systems - membrane redox complexes identified by their common name and different colours - carry out the oxidation of quinols (QH_2_) reduced by dehydrogenases. Besides oxygen (O_2_), nitrogen compounds can function as electron acceptors for the oxidation of dehydrogenases (dotted arrow), quinols and cytochrome *c* (dashed dark blue arrows), in reactions catalysed by enzyme complexes such as *Nrf* nitrite reductase [32], which are included within the N-metabolism system. Thick black arrows indicate electron transport in aerobic bacteria and mitochondria. Blue arrows indicate other electron transport pathways of facultatively anaerobic bacteria. **B - Pathways of mitochondrial bioenergetic evolution.** The bioenergetic systems illustrated in A are indicated by the coloured modules (with size proportional to their bioenergetic output) within the boxes representing the bioenergetic subset of each organism or organelle. Mitochondria of fungi and heterokont microorganisms differ from those of other eukaryotes for the presence of elements of N-metabolism. Representative taxa with fully sequenced genome are listed beneath each subset. The pathways of mitochondrial evolution are deduced by connecting these subsets with stepwise loss of a single bioenergetic system. Microorganisms underlined are symbionts or pathogens. Bacteria in embossed typeface have been proposed as ancestors or relatives of mitochondria (see Table S1 in File S1 for specific references). Dark brown arrows A and B indicate the pathways leading to fungal mitochondria. The pathway between the Rickettsia subset and that of mitochondria (dashed arrow) can be discounted, since the symbiotic event occurred only once [1], [5], [6], [10], [48]. * indicates the subset from which other pathways depart (Figure S1 in File S1).

This work follows a novel approach to identify proto-mitochondrial relatives among extant organisms by focusing on the bioenergetic systems that are common between mitochondria and bacteria. An enormous increase in bioenergy production constitutes the major advantage gained in the endosymbiotic event that led to the evolution of eukaryotic cells [2]. Consequently, the mitochondrial systems that generate most cellular bioenergy must define the minimal bioenergetic capacity of proto-mitochondria. Whereas aerobic α-proteobacteria such as *Pelagibacter* present the same two bioenergetic systems of animal mitochondria [4], [12], other proposed ancestors of mitochondria such as *Rhodospeudomonas palustris* [6]–[8] possess four additional bioenergetic systems in their terminal respiratory chain (Fig. 1A). These systems are characteristic of bacteria living under anaerobic or micro-oxic conditions, exploiting also bioenergy-producing elements of N-metabolism which are partially retained in some eukaryotic microorganisms [10], [22], [23]. It is thus likely that the current bioenergetic portfolio of mitochondria has evolved from a larger genomic endowment of bioenergetic systems which has been reduced via sequential loss.

We have reconstructed the possible pathways of this sequential loss leading to the bioenergetic systems of current mitochondria by evaluating all the genomes of α-proteobacteria which are currently available. Results obtained with complementary approaches then converged in indicating that methylotrophic α-proteobacteria could be the closest living relatives to proto-mitochondria, while excluding the majority of bacteria previously proposed as mitochondrial relatives.

## Results and Discussion

### 1.1 Reconstructed pathways of bioenergetic evolution of bacteria into mitochondria

The bioenergetic capacity of mitochondria has been instrumental in the evolution of eukaryotic cells and complex life forms [1]–[3]. It is generally assumed that proto-mitochondria had an aerobic energy metabolism equivalent to that of today's mitochondria [1], [4], [12], with the central part of the respiratory chain consisting of ubiquinol-cytochrome *c* reductase (the cytochome *bc*_1_ complex) and a single terminal oxidase, cytochrome *aa_3_* oxidase (Fig. 1A). However, geophysical evidence indicates that proterozoic oceans were essentially anoxic during the period in which the eukaryotic cell evolved [24]. Consequently, it is likely that proto-mitochondria were adapted to different levels of environmental oxygen, exploiting also the terminal oxidases of facultatively anaerobic bacteria to obtain bioenergy [10]. For example, *Rhodopseudomonas* strains possess cytochrome *bd* and *bo* ubiquinol oxidases [25], [26], plus an additional cytochrome *c* oxidase of the *cbb_3_* type [27] (Fig. 1B). Endocellular parasites have the *bd* ubiquinol oxidase either alone (in several species of *Rickettsia* [28]) or together with *cbb_3_* oxidase (in *Midichloria mitochondrii* [21]). Other organisms, moreover, possess proteins of the anaerobic bioenergetic process of denitrification, which are found also in mitochondria of fungi that can adapt to anaerobiosis [10], [23], [29].

Fungi and heterokont protists additionally possess an assimilatory nitrite reductase which is involved in ammonia fermentation, *NirB* fused with *NirD* [23], [29] – hereby defined as *NirBD*. In some bacteria, this NAD(P)H-dependent enzyme forms part of the nitrogen cycle that enables their growth from the oxidation of methane or ammonia, the oxidation of C1 compounds such as methanol (methylotrophy) and ammonification of nitrite [30]–[32]. Because various elements of this nitrogen cycle are associated with bioenergy production [23], [29]–[32], we have considered them within the broad bioenergetic system of N-metabolism (Fig. 1).

The metabolic versatility of current bacteria suggests that the ancestors of α-proteoproteobacteria had six bioenergetic systems from ubiquinol to oxygen (Fig. 1B), like diverse extant bacteria (Table S1 in File S1). To deduce the pathways of differential loss that led to the reduced subset of current mitochondria, we have developed a model based upon the bioenergetic systems coded in all available genomes of α-proteobacteria, including those we have recently sequenced (*Asaia platicody* and *Saccharibacter sp*. [22]). For parsimony, we allowed only single-step connections between the various subsets, thus obtaining two alternativepathways which direcly lead to the subset of bioenergetic systems that is present in contemporary mitochondria of fungi and protists (Fig. 1B, cf. Fig. S1 in File S1). Pathway A stems from the subset present in predatory *Micavibrio* [19] and also *Beijerinckia indica*, a metabolically versatile organism closely related to methylotrophs [33] which has been shown to possess several proteins strongly related to their mitochondrial homologues [8]. Alternative pathway B originates from the subset present in some *Magnetospirillum* species and two Rhodobacterales (Fig. 1B): *Roseobacter litoralis*, which retains a functional photosynthetic apparatus, and *Maricaulis maris*, which has a dimorphic biological cycle. The loss of N-metabolism from the *Micavibrio*/*Beijerinckia* subset leads to the subset of *Rickettsia* [28] and *Wolbachia* organisms which retain the *bd* ubiquinol oxidase system (Fig. 1B). The loss of this bioenergetic system would also lead to the subset of metazoan (but not fungal) mitochondria, a possibility considered unlikely in view of the unique symbiotic event producing mitochondria [1], [2], [10]. Moreover, it occurs in related species of the same Rickettiales order (Fig. 1B) and other taxa, for example within the *Bartonella* genus (Fig. S1 in File S1), suggesting phenomena of convergent evolution.

### 1.2 Testing the alternative pathways for mitochondrial bioenergy evolution

So, comparative genomic analysis has allowed a reconstruction of two possible reductive pathways in the bioenergetic capacity of bacteria evolving into mitochondria (Fig. 1). How can we establish which of these pathways is most likely, and thus identify extant models for proto-mitochondria? Probabilistic approaches based upon the frequency of gene loss from each subset would not produce conclusive evidence, because of the biased phylogenetic distribution of available bacterial genomes. We have then carried out the classical phylogenomic approach of computing the overall relationships of the organisms in the model of Fig. 1B by using concatenated proteins that are common to most eubacteria (cf. Ref. [21]). Although the obtained trees could be globally consistent with the sequence of either pathway A or B, they did not offer discriminatory evidence in favour of one or the other, while consistently placing *Midichloria* and other Rickettsiales close to the mitochondrial clade. This tree topology has been reported before [1], [4], [5], [21] but is inconsistent with our new model of Fig. 1B and other evidence [1], as discussed above.

We next followed the alternative approach of exploiting the molecular diversity of key bioenergetic proteins, including their multiple duplication [34]. To enhance the discriminatory power of this approach, we have chosen proteins of energy metabolism that have a clear bacterial origin, but are encoded or located in different compartments of eukaryotic cells (cf. [34]). The hypothesis underlying our approach is that such diverse proteins, as well as their genetic clusters, would present transition forms between bacteria and mitochondria predominantly in those organisms that are close to the proto-mitochondrial lineage.

### 2. Molecular evolution of assimilatory N metabolism

**Figure 2:**
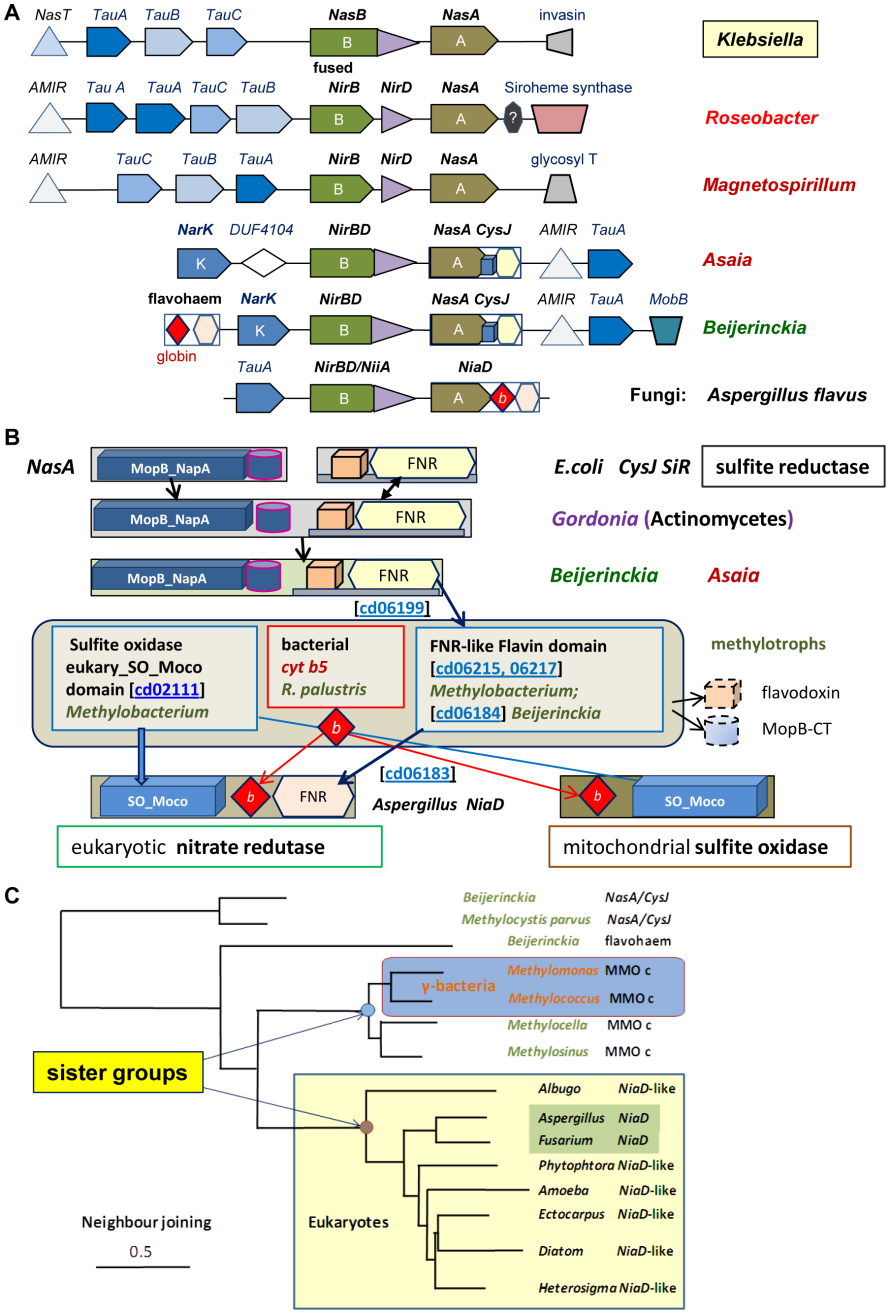
Graphical representation of assimilatory nitrate reduction in protists and α-proteobacteria. **A – The diagram shows the gene clusters of assimilatory, NAD(P)H-dependent nitrate reduction in bacteria and eukaryotes.** The various elements of *Nas* operon of *Klebsiella* [36] and the *NiiA-NiaD* operon in fungi [35] are colour coded as indicated in the quandrant on the top right. **B – Possible molecular evolution of fungal** ***NiaD*** **nitrate reductase.** Each domain is identified by a specific symbol - see the text for details. **C – Representative distance tree of various proteins containing the bacterial FNR-like conserved domain.** The tree was obtained with Neighbour Joining (maximal distance 0.9) using the DELTABLAST program [80] with methane monooxygenase subunit c of *Methylocella silvestris* (MMOc, Accession: YP_002361598) as query. This reductase subunit of methane monooxygenase contains a FNR-like domain similar to that of assimilatory nitrate reductases [43] lying in a sister group as indicated.

The first bioenergetic system we considered is N metabolism, the presence or absence of which sharply determines the pathways leading to the mitochondria of fungi and metazoans (Fig. 1B). As mentioned above, fungi and heterokonts possess the assimilatory, NAD(P)H-dependent nitrite reductase *NirBD* [35], a cytosolic enzyme which is common among facultatively anaerobic γ-proteobacteria such as *Klebsiella*, where it was originally called *NasB* [36]. Structurally, *NirBD* is characterised by the fusion of the small protein *NirD* - belonging to the Rieske superfamily of Fe-S proteins coordinated by histidines and cysteines [37] - at the C-terminus of the *NirB* protein, which catalyses the reduction of nitrite and is structurally related to sulfite reductase (*SiR*) [38]. Interstingly, the distribution of *NirB* is restricted to a relatively narrow group of facultatively anaerobic bacteria [38], [39], but that of *NirBD* is much narrower (Table 1). After finding *NirBD* in the genome of *Asaia*, we detected only ten homologus genes among α-proteoproteobacteria – compared with over one hundred in fungi (Table 1), all arranged in similar gene clusters comprising a regulator, nitrate transporters and an assimilatory nitrate reductase. The gene clusters are related to the *Nas* operon of *Klebsiella* (Fig. 2A), with its most compact version being present in fungi and Oomycetes [35].

**Table 1:** Elements of N-metabolism that are shared by bacteria and eukaryotes.

Taxonomic group and organism	NAD(P)H dependent, assimilatory			PQQ-dehydrogenase
	*NirB*	*NirBD*	*NiaD*-related proteins	*MxaF*
**methanotrophs & methylotrophs**				
***Methylocystis*** ** sp. SC2**	**yes**		**1 domain**	**yes**
***Methylocystis parvus***			**precursor & 1 domain**	**yes**
***Methylosinus trichosporium*** ** OB3b**	**yes**		**1 domain**	**yes**
***Methylosinus sp.*** ** LW4**			**1 domain**	**yes**
***Methylocella silvestris*** ** BL2**	**yes**		**1 domain**	**yes**
***Beijerinckia indica*** *		**yes**	**precursor & 2 domains**	**yes**
***Microvirga*** ** sp. WSM3557**	**yes**			**yes**
***Methylobacterium extorquens*** ** DM4**			**3 domains**	**yes**
***Methylobacterium extorquens*** ** PA1**			**3 domains**	**yes**
***Methylobacterium extorquens*** ** AM1**			**2 domains**	**yes**
***Methylobacterium extorquens*** ** CM4**				**yes**
***Methylobacterium extorquens*** ** DSM 13060**				**yes**
***Methylobacterium nodulans*** ** ORS 2060**			**2 domains**	**yes**
***Methylobacterium populi*** ** BJ001**			**2 domains**	**yes**
***Methylobacterium radiotolerans*** ** JCM 2831**			**2 domains**	**yes**
***Methylobacterium mesophilicum*** ** SR1.6/6**			**2 domains**	**yes**
***Methylobacterium*** ** sp. GXF4**			**2 domains**	**yes**
***Methylobacterium*** ** sp. 88A**			**2 domains**	**yes**
***Methylobacterium*** ** sp. 4**–**46**				**yes**
***Xanthobacter autotrophicus*** ** Py3**	**yes**			**yes**
***Hyphomicrobium denitrificans*** ** 1NES1**	**yes**			**yes**
**Bradyrhizobiaceae**				
***Nitrobacter winogradskyi*** ** Nb-255**	**yes**			
***Nitrobacter hamburgensis*** ** X14**	**yes**			
***Nitrobacter hamburgensis sp. Nb-255***	**yes**			
***Oligotropha carboxidovorans*** ** OM4 & OM5**	**yes**			
***Rhodopseudomonas palustris*** ** BisA53**			**2 domains**	**yes**
***Rhodopseudomonas palustris*** ** BisB18**			**1 domain**	**yes**
***Rhodopseudomonas palustris*** ** TIE-1**			**2 domains**	
other 4 *Rhodopseudomonas palustris*			**1 domain**	
**Rhodospirillales**				
***Granulibacter bethesdensis*** ** CGDNIH1**	**yes**		**2 domains**	yes
***Commensalibacter intestini*** ** A911**	**yes**			
***Acidocella sp.*** ** MX-AZ02**	**yes**		**1 domain**	
***Acidiphilium multivorum*** ** AIU301**	**yes**			
***Acidiphilium cryptum*** ** & sp. PM**	**yes**		**1 domain**	
***Gluconobacter oxydans*** ** H24**		**yes**	**precursor & 2 domains**	
***Gluconobacter frateurii*** ** NBRC 103465**		**yes**	**precursor**	
***Gluconacetobacter oboediens*** ** 174Bp2**		**yes**	**precursor & 2 domains**	
***Acetobacter pasteurianus*** ** IFO 3283-01/32**		**yes**	**precursor**	
***Acetobacter aceti***		**yes**	**precursor & 1 domains**	
***Gluconacetobacter europaeus*** ** LMG 18494**		**yes**	**precursor**	
***Gluconacetobacter diazotrophicus*** ** PAI5**			**2 domains**	
***Acetobacter pomorum*** ** DM001**		**yes**		
***Acetobacter tropicalis NBRC 101654***		**yes**		
***Asaia platicody***		**yes**	**precursor**	
***Saccharibacter*** ** sp.**	**yes**		**2 domains**	
***Tistrella mobilis*** ** KA081020**–**065**	**yes**		**2 domains**	
***Azospirillum lipoferum*** ** 4B**	**yes**		**1 domain**	yes
***Azospirillum amazonense*** ** Y2**	**yes**			
***Azospirillum brasilense*** ** Sp245**	**yes**			
***Azospirillum*** ** sp. B510**	**yes**			
***Caenispirillum salinarum*** ** AK4**	**yes**			
***Thalassospira profundimaris*** ** WP0211**	**yes**			
***Thalassospira xiamenensis*** ** M-5**	**yes**			
***Magnetospirillum magneticum*** ** AMB-1**	**yes**			
***Magnetospirillum sp.*** ** SO-1**	**yes**			
***Magnetospirillum gryphiswaldense*** ** MSR-1**	**yes**			
**Rhodobacterales**				
***Oceanicola granulosus***			**1 domain**	
***Oceanicola*** ** sp. S124**	**yes**			
***Octadecabacter antarcticus*** ** 307**	**yes**			
***Paracoccus denitrificans*** ** PD1222**	**yes**			
***Roseobacter denitrificans*** ** OCh114**	**yes**			
***Roseobacter litoralis*** ** Och 149**	**yes**			
***Jannaschia sp.*** ** CCS1**	**yes**			
**Rhizobiales (other)**				
***Martelella mediterranea***			**precursor**	
***Aureimonas ureilytica***			**2 domains**	
***Sinorhizobium meliloti*** ** 1021**	**yes**		**2 domains**	
***Rhizobium leguminosarum bv. trifolii*** ** WSM1325**	**yes**			
other 32 Rhizobiales	**yes**			
**Sphingomonadales & Caulobacterales**				
***Novosphingobium nitrogenifigens***			**precursor**	
***Sphingomonas*** ** sp. 17**			**2 domains**	
***Sphingomonas*** ** sp. PAMC26621**			**1 domain**	
***Sphingopyxis alaskensis*** ** RB2256**	**yes**			
other 19 Sphingomonadales & 6 Caulobacterales	**yes**			
**total α-proteobacteria**	***ca.*** ** 100**	**10**	**12 precursors**	
**Eukaryotes**				
***Aspergillus fumigatus***		**yes**	**yes**	
other 130 fungi (predominantly Ascomycetes)		**yes**	**yes**	
***Ectocarpus silicosus***		**yes**	**yes**	
plus other 8heterokonts	(1 yes)	**yes**	**yes**	
***Aureococcus anophagefferens***		**yes**	**yes & 2 domains**	
***Acanthamoeba castellani***			**yes**	
**total Eukaryotes**	**1**	**140**	**141**	

Proteins closely related to *NirB*, *NirBD*, *NiaD* and *MxaF* are annotated as **yes**, or **precursor** in the case of *Nas/CysJ* nitrate reductase (Fig. 2). The column of *NiaD*-related proteins also lists the number of *NiaD* **domains** that have homologues proteins in each organism, e.g. flavohaem (cf. Fig. 2C).

*Its close relative *Beijerinckia mobilis* has been reported to grow on methanol and possess *MxaF*.

Among the bacteria associated with pathway A and B in Fig. 1B, only *Beijerinckia* possesses *NirBD* and its cognate gene cluster. *Roseobacter litoralis* and *Magnetospirillum* have *NirB w*ithin an operon similar to that of *Klebsiella* (Fig. 2A), whereas *Maricaulis* and *Micavibrio* do not have the same genes. This situation may well arise from secondary loss of metabolic traits in ecologically specialised organisms such as dimorphic *Maricaulis* and predatory *Micavibrio*. To gain further phylogenetic information, we then exploited the rare occurrence of *NirBD* and its associated nitrate reductase among α-proteoproteobacteria (Table 1), evaluating the molecular evolution of these modular proteins. The structure of *NirBD* is conserved in α-proteobacteria and eukaryotes [35] and apparently derives from *NirB* precursors that are present in methylotrophs such as *Methylocystis* (Fig. 2, cf. [35]).

Conversely, the structure of the large protein functioning as nitrate reductase in the *NirBD* gene cluster of α-proteobacteria resembles that of nitrate reductases from ancient bacteria such as *Gordonia*, which contains three redox modules formed by distinct domains. A typical Molybdenum cofactor-binding domain (Moco) occupies the N-terminus and includes a terminal part binding another molibdopterin cofactor as in *NapA* (periplasmic) and *NasA* (cytoplasmic) reductases [36]–[40]. This is followed by an intermediate domain homologous to the small redox protein flavodoxin (Fig. 2B top, cf. [38]). The C-terminus then contains a flavoprotein reacting with the electron donor NAD(P)H which, in combination with flavodoxin, forms a domain closely related to sulfite reductase *CysJ* of *E.coli* (represented by a grey bar in Fig. 2B, cf. [38]). The *CysJ-*related domain belongs to the superfamily of Ferredoxin Reductase-like domains, cd 00322 FNR-like [41], which includes also the C-terminal domain of fungal nitrate reductase, *NiaD* [35], [40].

Although the fine structure of the FNR-like domain indicates two separate subfamilies, cd01699 SiR_like for the *NasA/CysJ* bacterial proteins and cd06183 cytb5_reductase_like for the eukaryotic proteins, our detailed sequence comparison uncovered phylogenetic relationships with other bacterial proteins belonging to the same superfamily. In particular, flavodoxin reductases of the genus *Methylobacterium* and the reductase subunits of soluble methane monoxygenase [42], [43] (MMO, present also in close relatives of *Beijerinckia* such as *Methylocella*) were consistently found in sister clades to *NiaD* and related proteins of fungi, heterokonts and *Acanthamoeba* (Fig. 2C and Table 1). Moreover, the flavohaem oxidoreductase of *Beijerinckia* (accession YP_001833084), which contains a cytochrome *b*-related globin followed by a FNR-like domain, was found in an intermediate position between the *NiaD*-containing clade and the *NasA-CysJ* reductases of *Beijerinckia* and *Methylocystis parvus* (Fig. 2C). Notably, the gene of this protein is located at the beginning of *Beijerinckia* nitrate assimilation operon (Fig. 2A). Its Nitric Oxide dioxygenase activity is also similar to that of the hybrid nitrate reductase of microalgae from the heterokont group, e.g. *Chattonella subsalsa* (protein NR2-2/2HbN, accession: AER70127), which possess both a cytochrome *b*_5_ and a globin in the intermediate domain [44]. These flavoproteins, therefore, could be considered transition forms between *NapA/CisJ* reductases and eukaryotic assimilatory nitrate reductases.

In further support of the modular similarity between bacterial and eukaryotic NAD(P)H-dependent nitrate reductases, we have found that the Moco domain of *NiaD*-like eukaryotic proteins is present also in the sulfite oxidase of methylotrophs such as *Methylobacterium mesophilicum* and *extorquens* (accession: WP_010685750 and WP_003602739, respectively - Table 1 and Fig. 2B). Moreover, the genome of *Methylobacterium extorquens PA1* encodes a protein that is partially similar to bacterial cytochrome *b*_5_ (accession: YP_001638730), which is present only in *Rhodopseudomonas palustris* among α-proteobacteria (Fig. 2B and data not shown). Consequently, all three functional domains of eukaryotic assimilatory reductases have homologous proteins in extant α-proteobacteria, particularly among those with methylotrophic metabolism, as indicated by the presence of the signature methanol dehydrogenase *MxaF* [45] (Table 1). Hence, our data suggests that *NasA-CisJ* reductases of *Beijerinckia* and acetic acid bacteria, e.g. *Asaia*, represent the likely **precursors** of eukaryotic, *NiaD*-related nitrate reductase (Table 1 and Fig. 2B,C). The parallel evolution of mitochondrial sulfite oxidase, which shares the same cytochrome *b*_5_ and Moco domains with eukaryotic assimilatory nitrate reductases (Fig. 2B, cf. [38], [40]), underlines the intersection of this molecular reconstruction with the evolutionary trajectory of proto-mitochondria.

### 3. Evolution of *COX* genes and proteins from bacteria to mitochondria

To test alternative evolutionary pathways for mitochondria (Fig. 1B) we next studied the cytochrome *c* oxidase of *aa_3_*-type (also called *COX*), which appears to be the most common terminal oxidase in extant α proteobacteria (Fig. 1 and Table S1). In eukaryotes, this enzyme complex is embedded in the inner mitochondrial membrane, combining catalytic subunits of bacterial origin with various nuclear-encoded subunits of unknown function. Although all *aa_3_*-type oxidases are of type A according to the classification of heme-copper oxygen reductases [26], the complexity of their gene clusters has not been considered before. Here, we have analysed in depth this complexity for it provides valuable phylogenetic information. Various aspects of our analysis are presented below in the following order: 1, diversity of *COX* operons; 2, evolution of *COX* operons; 3, possible *COX* operons of proto-mitochondria; 4, evolution of the molecular architecture of *COX*3; 5, phylogenetic distribution of *COX* operons.

#### 3.1 Diversity of *COX* operons

**Figure 3:**
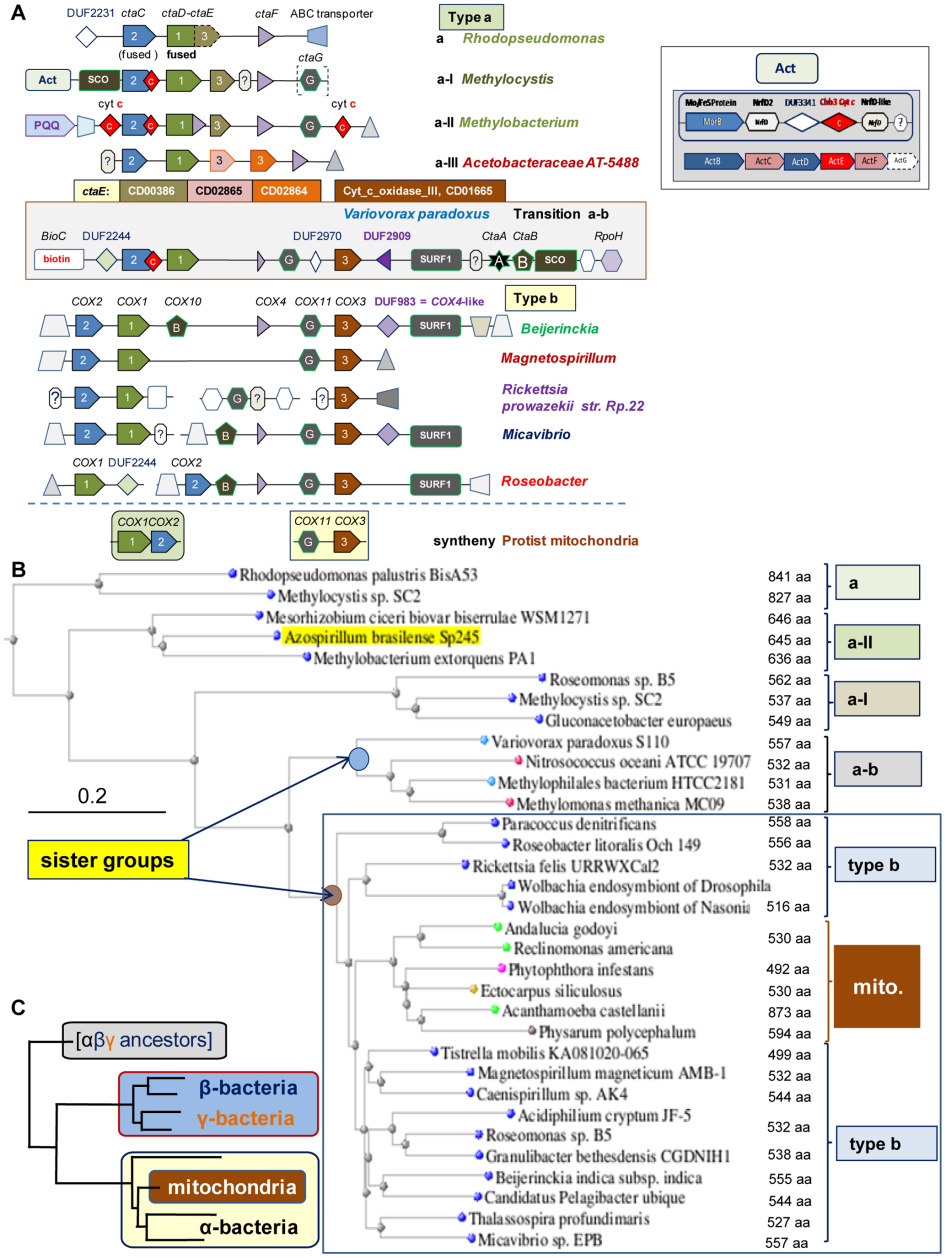
α-proteobacteria have different types of *COX* operons and catalytic subunits of *aa_3_* oxidase. **A – Graphical representation of *aa_3_* oxidase gene clusters.** The different *COX* clusters of α-proteobacteria are classified by considering gene sequence variations and the features of flanking genes (see also “Classification of bacterial *COX* operons” in File S1). Specific graphical symbols identify *COX* subunits as indicated; other types of proteins are labelled as follows: white hexagon, enzyme working with RNA or DNA; red diamond with enclosed c, cytochrome *c* type protein; truncated triangle pointing left, ABC transporter/permease; grey sharp triangle, transcription regulator; PQQ, PQQ-dependent dehydrogenase; white diamond, protein belonging to a DUF family [41], e.g. DUF983; question mark within hexagon, completely unknown protein. Note that *SURF1* (Surfeit locus protein 1) and *SCO* (Synthesis of cytochrome *c* oxidase) are also involved in the biogenesis of oxidases. Distance between genes is arbitrary. *COX* operon **type a-I** is attached to a *Nrf*-like gene cluster, also called Alternative Complex III or Act [50], containing two homologues of the membrane subunit *NrfD* (called *NrfD2* and *NrfD*-like here, as shown at the side of the figure). The synthenic diads of protist mitochondria [48] are shown below the blue line. Each of the recognised subfamilies of *COX*3 [41] is represented by a different colour, as indicated in the middle of the illustration. **B - Representative distance tree of** ***COX*** **1 proteins.** The tree was obtained with Neighbour Joining (maximal distance 0.9) using the DELTABLAST program [80] with the *COX*1 protein of *Methylobacterium extorquens* PA1 (Accession: YP_001637594) as query. The group containing bacterial and mitochondrial proteins (mito.) is enclosed in the blue square. Protein length and type of *COX* operon are annotated on the right of the tree. **C – Simplified pattern of typical phylogenetic trees of** ***COX*** **1 proteins.**The tree is modelled to match distance trees of nitrate reductase (Fig. 2C) and *COX*1 (part B). Branch length is arbitrary.

We have initially undertaken a systematic analysis of the genomic diversity of *aa_3_*-type oxidases.The scrutiny of all the gene clusters containing proteobacterial *COX* subunits [46]–[51] suggests that they fall into three distinctive types of *COX* operons, which we called type a, b and a–b transition (Fig. 3A – see Table S2 and “Classification of bacterial *COX* operons” in File S1 for a detailed account of this classification). *COX* operon type a is divided in four subtypes on the basis of *COX*1 length and diverse adjacent genes (Fig. 3). These subtypes form coherent clades in the phylogenetic trees of their *COX*1 subunit (Fig. 3B). Despite the variation in gene sequence, all *COX* operons appear to derive from the core structure of the *ctaA-G* operon of *Bacillus subtilis* [46]–[51] (Fig. 3A), which consists of the catalytic subunits *ctaC* and *ctaD* (corresponding to mitochondrial *COX*2 and *COX*1, respectively) followed by the hydrophobic, non-catalytic subunit *ctaE* (corresponding to mitochondrial *COX*3) and *ctaF* (also called *COX*IV or *COX*4). Mitochondrial DNA (mtDNA) of eukaryotes generally encodes for *COX*1, *COX*2 and *COX*3 [48]. In bacteria, these principal subunits are often combined with proteins for the assembly of the metal cofactors of the oxidase: *ctaA* (heme A syntase or *COX*15), *ctaB* (protoporphyrin IX farnesyl transferase, or *COX*10) and *ctaG* (Cu-delivery protein, or *COX*11).

Our systematic analysis of bacterial *COX* subunits has revealed a novel fusion between *COX*1 and *ctaF*/*COX*4 (Fig. S2 in File S1). This fusion appears to be restricted to *COX* operon type a-II (Table S2 in File S1 and Fig. 3A) that often contains Pyrroloquinoline quinone (PQQ)-dependent dehydrogenases such as methanol dehydrogenase related to *MxaF* (Fig. 3A). *COX*4 is broadly related to the *ctaF* subunit, which is the least conserved in the *caa_3_*-type oxidase of *Thermus* and *Bacillus* [47] but can be recognized as part of Cyt_c_ox_IV (pfam12270 [52]). However, the diverse forms of short hypothetical proteins that intermix with *COX* subunits (Fig. 3A) are generally not recognized as members of this family in BLAST searches, due to the wide variation in their size and sequence [47]. Therefore, we have developed a method that quantifies the sequence similarity with the *COX*IV proteins from *Rhodobacter* [53] and *Thermus* [47], [54], for which the 3D structure is available (see Fig. S2 in File S1 and its legend for details). Strong sequence similarity with these *COX*4 proteins was found in the C-terminal extension of bacterial *COX*1 proteins that are 630 to 670 aa long, as well as in mitochondrial *COX*1 of the pathogenic fungus, *Zymoseptoria tritici* [55] (Fig. S2A in File S1). We additionally identified the sequence signatures of *COX*4 in small proteins previously recognized as domain with unknown function (DUF [52]) families, namely DUF2909 and DUF983 (Figs. 3 and S3 in File S1). Morever, the C-terminal part of the mtDNA-encoded *COX*1 of ciliates, an ancient and diverse phylum of unicellular eukaryotes [56], shows some sequence similarity encompassing both transmembrane helices of *COX*4 proteins (Fig. S2A and B in File S1). Although this similarity is clearly weaker than that observed with bacterial *COX*1 proteins, it lies in a conserved region among ciliates (Fig. S2A in File S1 and data not shown) thereby suggesting that fusion of *COX1* with *COX4* might represent an additional trait shared by bacteria and mitochondria.

#### 3.2 Evolution of *COX* operons

The identification of *COX*4-like proteins has been combined with phylogenetic analysis to deduce the possible evolution of *COX* operons. The long proteins derived from the fusion of *COX*1 with *COX*3 (hereafter called *COX*1-3) seem to be the most distant from their mitochondrial homologues (Fig. 3B). These proteins are characteristic of *caa_3_* oxidases [46], [47], as well as of *COX* operon type a, which can therefore be considered the ancestral form of proteobacterial gene clusters for *aa_3_*-type oxidases (Fig. 3A). The differentiation into other types of COX operons can be evaluated also from the phylogenetic trees of the catalytic subunit *COX*1, the analysis of which has offered new evidence for discriminating the evolutionary pathways in Fig. 1B.

*COX*1 proteins fused with *COX*4 (see above) appear to follow the ancestral *COX*1-3 in phylogenetic trees and are always upstream of a major bifurcation in two large groups: one containing only proteins of *COX* operon a-b transition that are present in β- and γ-proteobacteria, and the other containing bacterial *COX*1 proteins of *COX* operon type b together with their mitochondrial homologues (blue square in Fig. 3B). Mitochondrial *COX*1 proteins cluster in a monophyletic clade that lies in sister position of closely packed bacterial sub-branches, especially that containing Rhodospirillales (Fig. 3B). This overall tree topology is consistently found with all methods, whereas the branching order within the group containing the mitochondrial clade may vary, depending upon the method and taxa used to construct the phylogenetic trees (Fig. 3B and data not shown). Nevertheless, it is noteworthy that all the proteins belonging to *COX* operon type b lie in the same group containing the mitochondrial clade, as exemplified in Fig. 3C. Hence, bacteria having only *COX* operon type b cannot be the ancestors of mitochondria. This exclusion encompasses the majority of extant α-proteobacteria, because the presence of other *COX* operons is restricted to a fraction of these organisms (Table S2 in File S1). We then needed additional information to identify which of the organisms containing multiple *COX* operons may be close to proto-mitochondria. To this end, we next moved to the analysis of *COX* proteins of unicellular eukaryotes.

#### 3.3. Possible *COX* operons of proto-mitochondria

Recently, *COX*11 and *COX*15 have been found in the mtDNA of Jakobida, an ancient lineage of protists, despite the fact that they are normally coded by nuclear DNA in eukaryotes [48]. The syntheny *COX*11*COX*3, as well as that of *COX*1 adjacent to *COX*2 (Fig. 3A), may be considered a relic of bacterial operons that has been retained in the mDNA of eukaryotes [48]. Are these cues pointing to the original *COX* operon(s) of proto-mitochondria?

**Figure 4:**
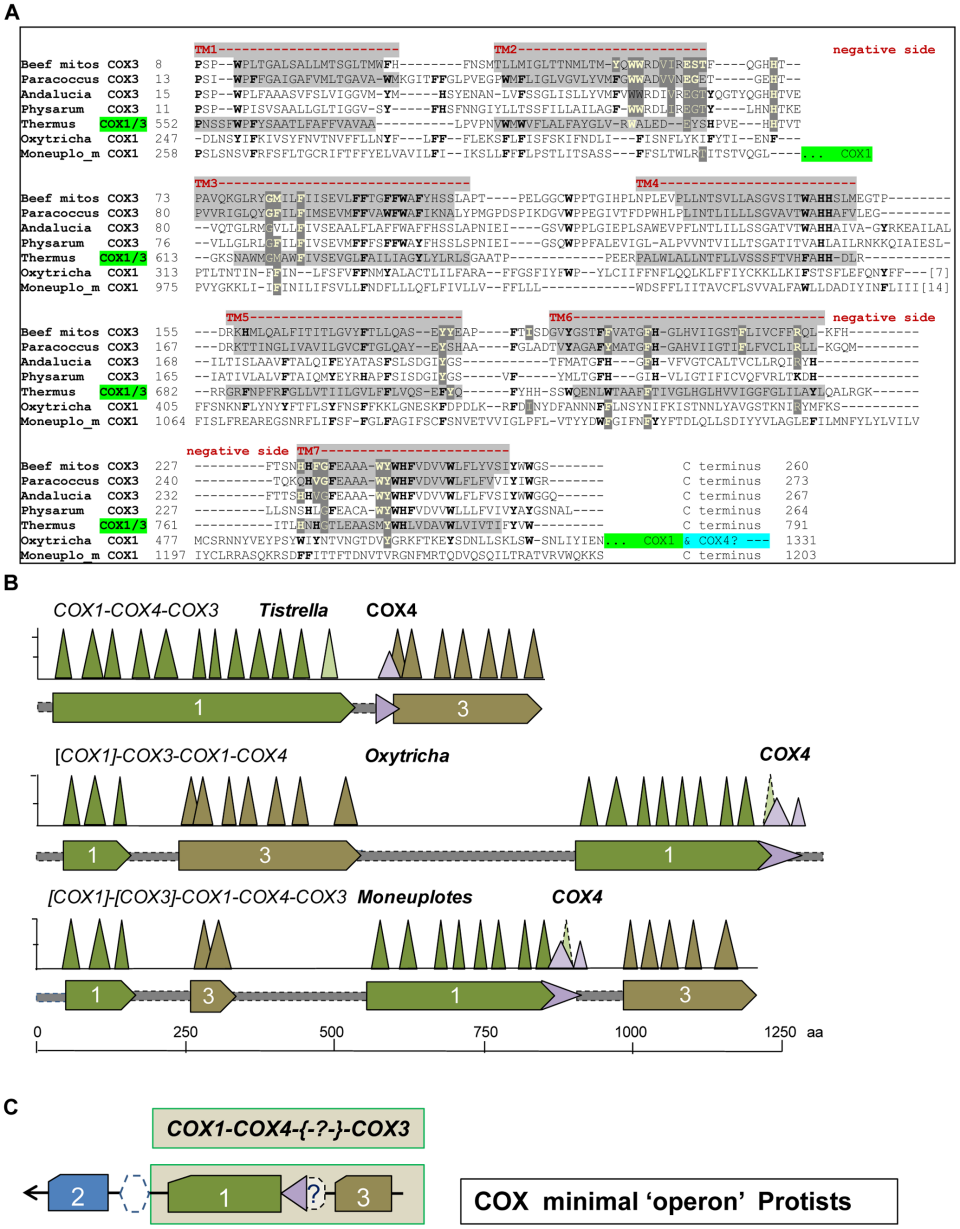
Analysis of the molecular architecture of *COX*3 in bacteria and protists. A – Alignment of bacterial and mitochondrial *COX*3 proteins. A set of aligned *COX*3 sequences from bacteria and protists was initially obtained from the DELTABLAST option of multiple alignment and subsequently implemented manually following data available from the structure of beef [59], [60], *Paracoccus* [61] and *Thermus* [54] *aa_3_* oxidase. Residues that bind phospholipids with either H or π bonds [60] are in yellow character and highlighted in dark grey, while those conserved are in bold character. Light grey areas indicate transmembrane helices (TM). **B – Graphical representation of** ***COX*** **1-3 fused proteins.** The hydrophobic peaks in the hydropathy profile of the proteins, which was obtained using the program WHAT [81] with a fixed scanning window of 19 residues, is represented by the sharp triangles, that are commensurated to the peak height (maximum in the hydrophobicity profile) and width of the predicted TM [81], which closely correspond to those observed in 3D-structures [47], [54], [61]. **C – Deduced sequence of the “minimal”** ***COX*** **operon of protists.** The arrangement of *COX* genes essentially corresponds to the core sequence of a *COX* operons of type a (cf. Fig. 3) but in the reverse order of transcription. Dashed symbol represents a protein that may intermix with other *COX* subunits such as a *COX*4-like (Fig. S2 in File S1).

To answer this question, we searched the available mtDNA genomes of unicellular eukaryotes. Mitochondrial DNA normally contains separate genes for *COX*1, *COX*2 and *COX*3 [48] except for aerobic ciliates, in which *COX*3 appears to be missing [56], [58]. However, we have recognized the sequence signatures of the *COX*3 protein within the very long *COX*1 of the hyphotrichous ciliate, *Oxytricha* [56] (Fig. 4). The *COX*1 protein of another hyphotrich, *Monoeuplotes minuta* [58], appears to contain a split version of *COX*3 having its initial two transmembrane helices separated from the subsequent 5-transmembrane helices domain by the major part of *COX*1 (Fig. 4). The mtDNA of ciliates often contains split genes [56], [58], but in this case an ancestral splitting of *COX*3 must have been subsequently intermixed with the *COX*1 gene. The alternative possibility would be that *COX*3 splitting may reflect a fusion between precursors of mitochondrial *COX*3, since in *Monoeuplotes* it occurs within the region joining the two transmembrane domains which form the V-shaped structure of the protein [53], [59]-[61].

In any case, the novel identification of a *COX*3-like protein embedded within the long *COX*1 gene of unicellular eukaryotes (Fig. 4) suggests that the primordial form of such a chimaeric gene was a *COX*1-3 protein equivalent to those of bacterial *COX* operons of type a. By considering the gene order in ciliate mtDNA [56], [58], we have deduced the possible sequence of the “minimal” *COX* operon that might have been present in the ancestors of ciliate mitochondria (Fig. 4C). The gene sequence closely resembles the core structure of a *COX* operon of type a - in the opposite order of transcription (cf. Fig. 3A and 4C) - and is clearly different from the sequence of *COX* operon type b (Figs. 3A and S3 in File S1). In view of the consensus that a single event of symbiosis originated all mitochondria [1]–[10] and considering the presence of *COX*11*COX*3 syntheny in Jakobide mitochondria [48], a feature characteristic of *COX* operon type b (Figs. 3 and S3 in File S1), we surmise that proto-mitochondria possessed two different *COX* operons: one of type a and another of type b. Differential loss of either operon might further explain some differences in the mtDNA-coded proteins of ciliates and other unicellular eukaryotes, as well as the different types of accessory subunits of their bioenergetic complexes [1]. Of note, phenetic analysis sustains the similarity between the *COX* gene sequence of protists and bacterial *COX* operon of type a-II, in particular those lacking an isolated *COX*4 as in *Methylobacterium extorquens* PA1 (Table S3 in File S1).

#### 3.4 Evolution of the molecular architecture of *COX*3

**Figure 5:**
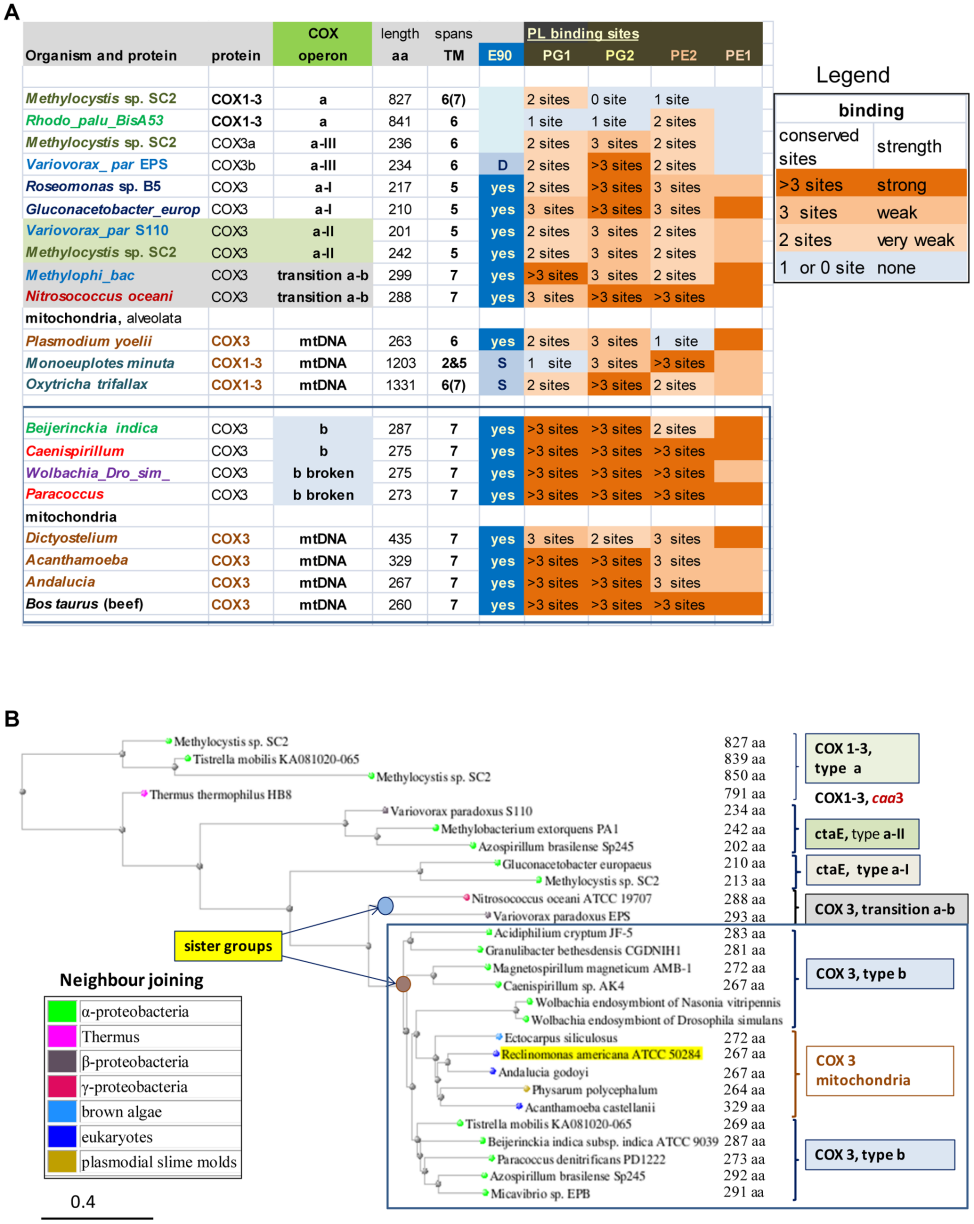
Structure-function features of *COX*3 gradually evolved from bacteria to mitochondria. **A – Heatmap for the strength of phospholipid binding by *COX*3 proteins.** The table summarises the molecular features of PL-binding sites (residues) in aligned *COX* 3 proteins (Table S4 in File S1); it is colour mapped according to the number of conserved sites to represent the increasing PL-binding strength along bacterial and mitochondrial protein sequences, as indicated by the legend on the right of the table. PL-binding is considered weak when less than 3 sites are conserved for each PL, the nomenclature of which is taken from Ref. [60]. PE, phosphatidyl-ethanolamine; PG, phosphatidyl-glycerol. The list includes conserved amino acids corresponding to E90 in beef *COX*3, which lies near bound PL modulating oxygen entry into the catalytic site of the oxidase [60]. Abbreviations for organisms are*: Rhodo_palu_BisA53*, *R. palustris* BisA53; *Variovorax_ par*, *Variovorax paradoxus*; *Methylophi_bac*, *Methylophilales bacterium* HTCC2181; *Wolbachia_Dro_sim_, Wolbachia* endosymbiont of *Drosophila simulans*. **B - Representative distance tree of** ***COX*** **3 proteins.** The tree was obtained as described in the legend of Fig. 3B, using as a query the C-terminal region of the *COX*1-3 protein from *R. palustris* BisA53 (Accession: YP_782773, residues 550 to 841) that aligns with bacterial and mitochondrial *COX*3 (Fig. 3B Fig. 4A). The group containing bacterial proteins from *COX* operon type b and their mitochondrial homologues is enclosed in a blue square as in Fig. 3B.

In the 3D structures available for cytochrome *c* oxidases, the initial two transmembrane helices of the 7- helices *COX*3 protein that is present in mitochondria and bacterial *COX* operon type b (Fig. 3A) are involved in the binding to membrane phospholipids (PL) [53], [59]–[61]. The tight binding of two specific forms of these PL to mitochondrial *COX*3 appears to modulate the entry of oxygen into the binuclear catalytic centre of the enzyme [60]. PL-binding residues are present also in other parts of the *COX*3 protein that are common to all its forms and tend to be conserved [59]–[62]. Here, we have evaluated the amino acid substitutions of the PL-binding sites in *COX*3 (Table S4 in File S1) by translating residue varation into PL-binding strength (Fig. 5A). The results of this analysis are consistent with the phylogenetic trees of *COX*3, in which a major bifurcation separates the β- and γ-proteobacterial proteins from those of α-proteobacteria that are grouped together with mitochondrial *COX*3 (Fig. 5B). The overall tree topology of *COX*3 proteins thus matches that of *COX*1 proteins, even if the internal branching of α-bacteria with the mitochondrial clade appears to be different (Fig. 5B cf. Fig. 3B).

Quantitative evaluation of the PL-binding strength further refines the evolutionary relationship among *COX*3 proteins. First, it shows that the 5-helices form of the protein belonging to *COX* operon type a-II occupies an intermediate position between ancestral *COX*1-3 and the 7-transmembrane form of *COX*3 (Fig. 5A). Secondly, it allows the comparison with the highly divergent sequence of ciliate *COX*3 embedded within *COX*1 (Fig. 4), which shows a PL-binding strength lying mid-way between that of *COX*3 proteins of type a-II operon and those of other protists (Fig. 5A and Table S4 in File S1). Finally, bacterial *COX*3 of *COX* operon type b has essentially the same PL-binding strength as that of mitochondrial *COX*3 (Fig. 5A and Table S4 in File S1), thereby weakening the structural and phylogenetic significance of variable inter-group branching between α-bacterial and mitochondrial *COX*3 sequences (Fig. 5B and data not shown).

#### 3.5. Phylogenetic distribution of *COX* operons

To acquire further information for differentiating the pathways of mitochondrial evolution in Fig. 1B, we studied the phylogenetic distribution of diverse *COX* operons. The vast majority of Rhodobacterales, Sphingomonadales and Caulobacterales, together with unclassified α-proteobacteria such as *Micavibrio* and the SAR11 clade - which we include here under the generic label of ‘pan-Thalassic’- possess only *COX* operons of type b. This implies that *Roseobacter* and *Micavibrio* cannot be related to the ancestors of mitochondria, as for *Pelagibacter* and similar marine organisms.

**Figure 6:**
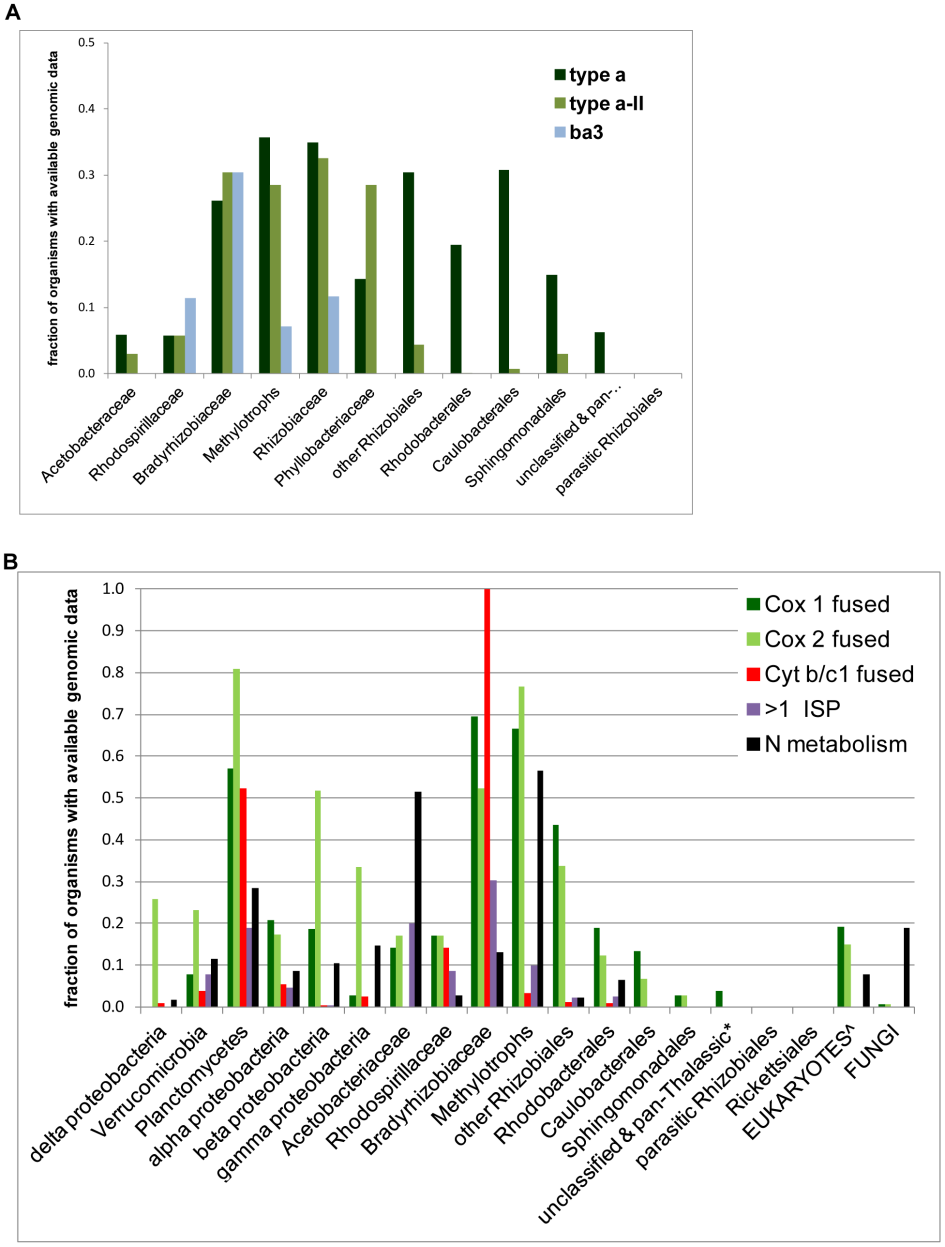
Taxonomic distribution of bioenergetic systems in bacteria. **A – Distribution of *COX* operon types in major families of α-proteobacteria.** The frequency of each type of *COX* operon was normalised to the number of α-proteobacterial organisms with genomic data that are currently available (from NCBI resources http://www.ncbi.nlm.nih.gov/taxonomy/- accessed 14 March 2014) [50]. See Table S2 in File S1 for a detailed list of the taxonomic distribution of diverse *COX* operon types. The definition ‘pan-Thalassic’collects together organisms of the SAR clade with *Magnetococcus, Pelagibacter* and *Micavibrio*. **B. -Distribution of fused proteins and N-metabolism elements along diverse bacterial lineages.** Fused proteins were identified with the combined resourses of NCBI and the Protein Family website (PFAM 27.0 - http://pfam.sanger.ac.uk/ [52]). Multiple forms of ISP were counted as >1 ISP. Taxa are arranged according to their approximate phylogenetic position considering also metabolic features (cf. Refs [5], [31]). For each group, the frequency is normalized as in A. Eukaryotes (∧) include amoebozoa, ciliates and heterokonts. N-metabolism encompasses: methane monooxygenase, ammonia monooxygenase, nitrite oxidoreductase, *Nirf* nitrite reductase and its homologues in COX operon type a-I (Fig. 3A), ammonia oxidation and anaerobic ammonia fermentation [30], [32].

On the other hand, 40 α-proteobacterial organisms and several β-proteobacteria combine *COX* operon type b with a type a-II operon, the phylogenetic distribution of which is similar to that of *ba_3_* oxidases [26] (Fig. 6A). Conversely, *COX* operon type a-I has the broadest phylogenetic distribution among all types of *COX* operon, encompassing taxonomic groups beyond the *phylum* of proteobacteria such as Planctomycetes [50]. Indeed, the *Nrf*-like gene cluster that is associated with this *COX* operon was originally discovered in ancient eubacteria including Planctomycetes [63]. Although the functional implications of the combination of a *Nrf*-like operon with a *COX* gene cluster remais unknown, we are intrigued by the possibility that the overall gene sequence would produce a compact electron transport chain from quinol, or products of N metabolism, to oxygen [32], [50]. Consequently, *COX* operon type a-I would represent the ultimate bioenergetic connection between cytochrome *c* oxidase and N metabolism, a fundamental concept in our approach to discern mitochondrial evolution (Fig. 1).

### 4. Phylogenetic distribution of N metabolism and fused proteins in bacteria and mitochondria

To explore the phylogenetic dimension of the connection between *COX* operons and elements of N metabolism, we studied the taxonomic distribution of *NrfD* and other key elements of the N cycle in conjunction with that of fused subunits of *aa_3_*-type oxidases (Fig. 6B). Indeed, *COX* operon type a-I invariably contains *COX*2 fused with a *c*-type cytochrome (Figs. 3A), a fusion which is frequently present also in other *COX* operons (Fig. 3A and Fig. S3 in File S1). Fusion between catalytic subunits of bacterial heme-copper oxidases has been noted before [47], [64], but considered a nuisance for phylogenetic analyses [64]. However, it constitutes a relic of ancestral bacteria adapted to harsh conditions in which the compact structure of bioenergetic systems would have been advantageous [47]. Since we have now shown that fusion between *COX* subunits is present also in the mitochondria of unicellular eukaryotes (Fig. 4) and fungi such as *Phaeosphera* [57], we could consider them as potential relics of the evolutionary past of mitochondrial bioenergetics.

We therefore evaluated the frequency and phylogenetic distribution of fused *COX* subunits and also of the fused proteins that are present in the cytochome *bc*_1_ complex, the cytochrome *b* subunit of which has been previously reported to be fused with the cytochrome *c*_1_ subunit in *Bradyrhizobium* [65]. We found the same fusion in all members of the Bradyrhizobiaceae family plus some Rhodospirillales (Fig. 6B), as well as in Planctomycetes [66]. α-proteobacteria show the highest frequency of fused cytochrome *b* among proteobacterial lineages, thereby suggesting that this type of protein was present before the separation of β- and γ-proteobacteria. Conversely, many more β-proteobacteria possess fused *COX*2 proteins than α-proteobacteria (Fig. 6B).

Within α-proteobacteria, the distribution of fused *COX* and cytochrome *b* proteins follows a bell-shape profile along the likely evolutionary sequence of the taxonomic groups (Fig. 6B, cf. [5]). Some Sphingomonadales and Caulobacterales have fused *COX* proteins without possessing bioenergetic elements of N-metabolism (Fig. 6B). Parasitic Rhizobiales, Rickettsiales and pan-Thalassic organisms lack both fused bioenergetic proteins and elements of N-metabolism, in contrast with amoebozoa, fungi and heterokonts (Fig. 6B cf. Table 1). The absence of the above characters in parasitic and pan-Thalassic organisms could derive from their highly streamlined genomes. However, the high frequency of fused genes in other taxa does not correlate with genome size, since acetic acid bacteria, which have a comparatively small genome, show a higher frequency of fused *COX*2 proteins than, for instance, Rhodobacterales (Fig. 6). Our interpretation of the data presented in Fig. 6 is that fused bioenergetic proteins and elements of N metabolism are preserved together in phylogenetically ancient groups of α-proteobacteria, from which they have been passed to proto-mitochondria but then progressively lost along the differentiation of other α-proteobacteria. This implies that Methylotrophs, Bradyrhizobiaceae and several Rhodospirillales would be the oldest extant organisms of the α-proteobacterial lineage, and consequently close to the distal progenitors of proto-mitochondria.

The phylogenetic distribution and similar genomic arrangement of fused bioenergetic proteins (Fig. 6) raises the question as to whether they may derive from events of Lateral Gene Transfer (LGT), for example with Planctomycetes [67]. However, detailed analysis of the molecular architecture of cytochrome *b* proteins (M. Degli Esposti, unpublished data) and the overall consistency of distance trees of fused proteins with established phylogenetic relationships (Fig. 3) indicate that LGT events have minimally contributed to the observed distribution of fused bioenergetic proteins and their diverse genomic clusters.

### 5. A complementary approach: the molecular evolution of nuclear encoded ISP

**Figure 7:**
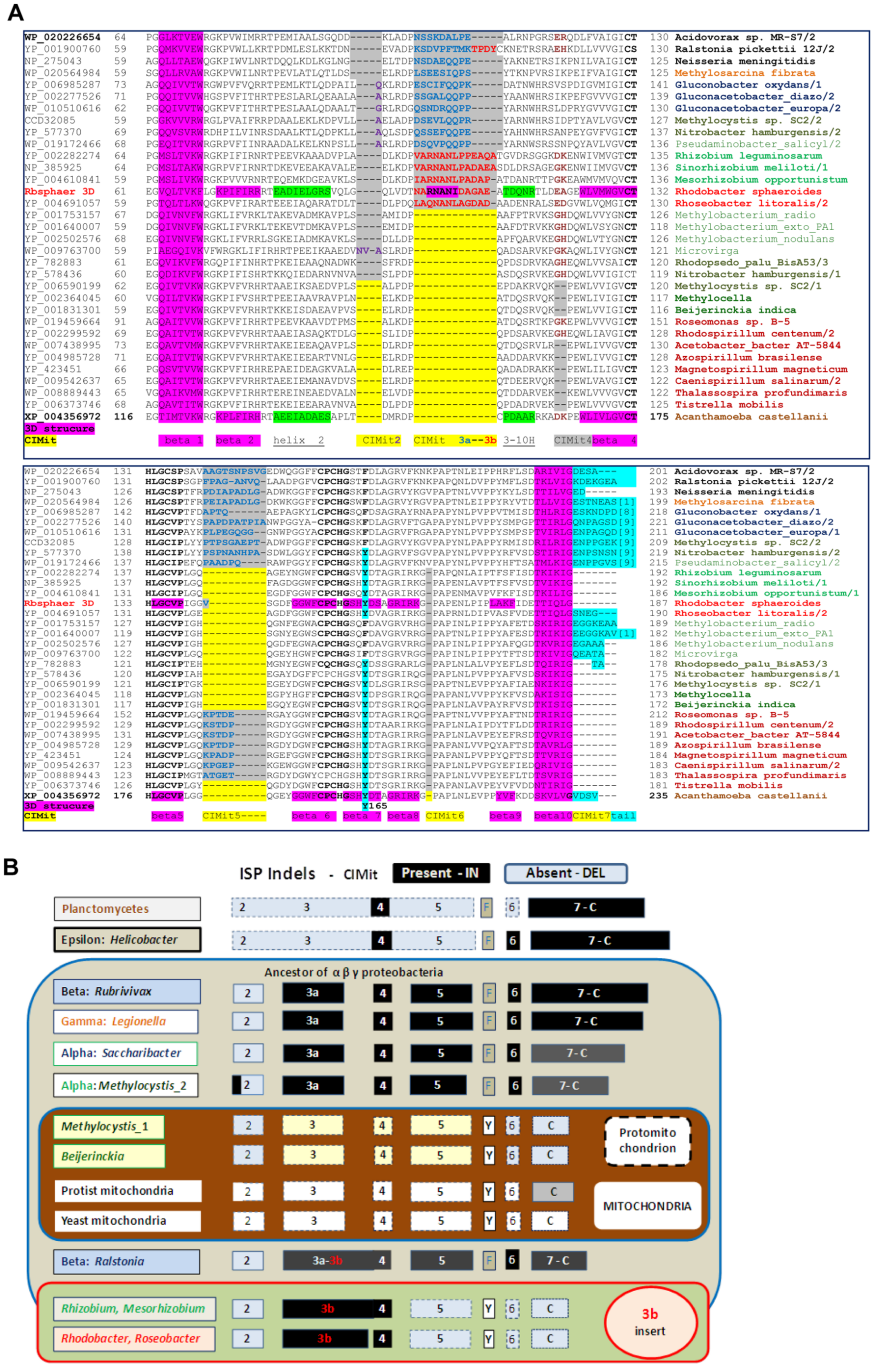
Molecular evolution of the Rieske subunit (ISP) of the cytochrome *bc_1_* complex. **A – Alignment of the ISP proteins from bacteria having various *COX* operons.** ISP sequences were selected from the organisms displaying multiple *COX* operons and also ISP forms (Table S2 in File S1 and Fig. 6). The alignment was manually refined using structural information, as detailed in Fig. S4 in File S1. This alignment shows only the catalytic core of the ISP from α-, β- and γ-proteobacteria, plus *Acanthamoeba* as the sole mitochondrial representative. See Fig. S4 in File S1 for a complementary alignment including the N-terminal transmembrane region and further information, including secondary structure elements (beta sheet in purple and alpha helix in green) and Conserved Indels vs. Mitochondria (CIMit). The accession codes of the proteins are shown on the left of each sequence block, while the organisms are listed on the right abbreviated as follows: Gluconacetobacter_diazo & _europa, *Gluconacetobacter diazotrophicus* PA1 5 & *europaeus,* respectively; Pseudaminobacter_salicyl, *Pseudaminobacter salicylatoxidans*; Methylobacterium_radio & _exto_PA1, *Methylobacterium radiotolerans* JCM 283 & *extorquens* PA1, respectively; Rhodopsedo_palu_BisA53, *R. palustris* BisA53; and Acetobacter_bacter AT-5844, *Acetobacteraceae bacterium* AT-5844. ISP1 indicates the ISP form that is present in the *petABC* operon. **B - Evolutionary pattern of the conserved indels in bacterial and mitochondrial ISP.** The molecular features deduced by the structure-based alignment of ISP proteins are rendered graphically following the numerical order of conserved indels presented in A and Fig. S4 in File S1. DELetions conserved in bacterial vs. mitochondrial ISP sequences are represented in pale blue boxes with black labels, whereas INserts with respect to mitochondrial sequences are represented in black boxes with white labels.

**Figure 8:**
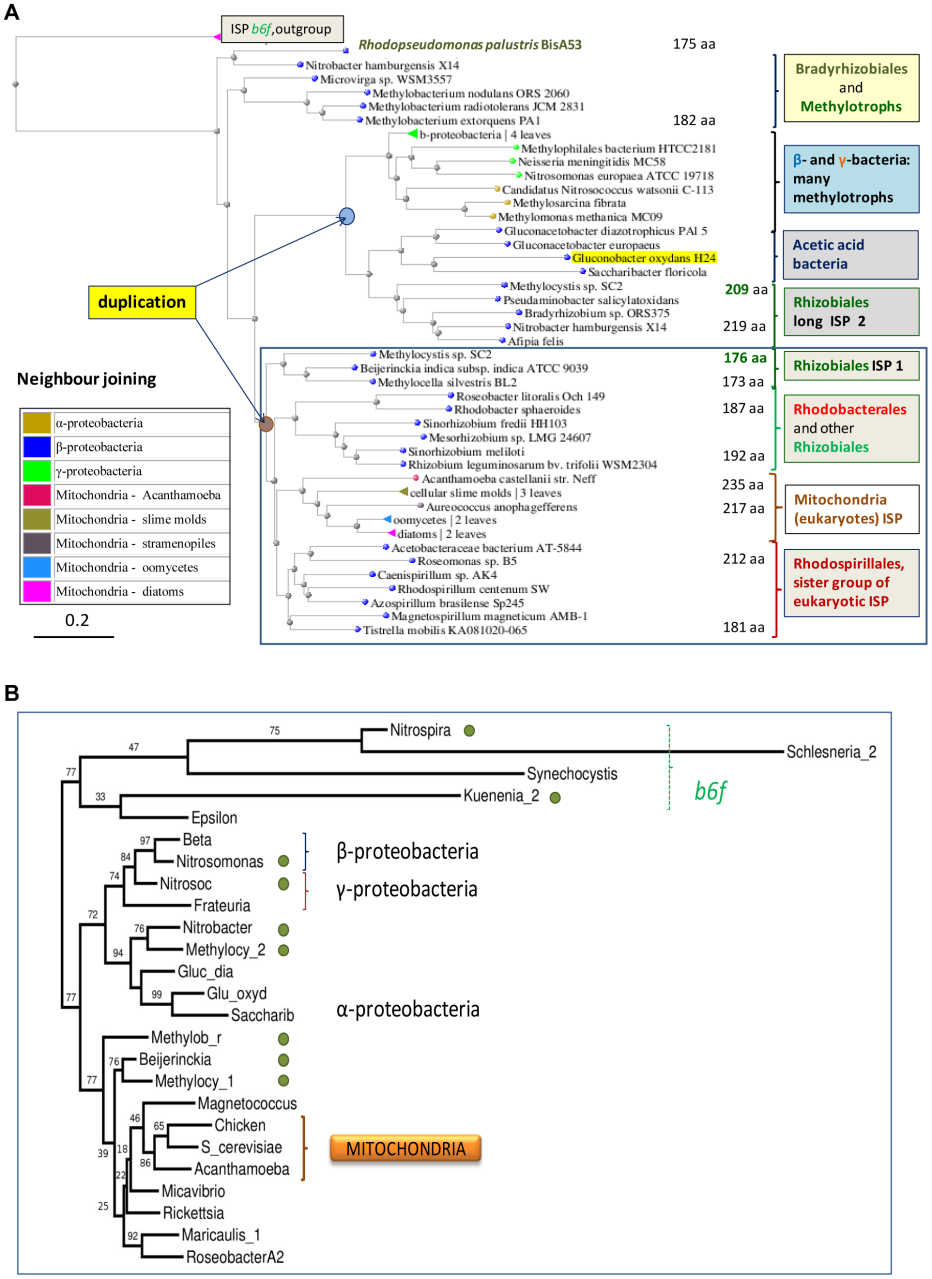
Phylogenetic relationships between diverse forms of ISP. **A – Distance tree encompassing proteobacteria and mitochondria.** The tree was obtained as described in the legend of Fig. 3B using the alignment of Fig. 7A and two ISP proteins from the *b_6_f* complex as outgroup (top). The group containing bacterial ISP1 proteins together with their mitochondrial homologes is enclosed in the blue square to highlight a likely ancestral duplication separating it from the group with ISP2. **B – Long distance phylogenetic relationships of bacterial ISP.** The phylogenetic tree (maximal likelyhood method) of ISP proteins was computed from the structure-based alignments in Fig. S4 in File S1. Th small green circle indicates ancient nitrogen or methylotrophic metabolism [29]–[32] (Fig. 6B). The dashed green bracket indicates the paralogue proteins belonging to the *b_6_f* complex. Other brackets indicate proteobacterial subdivisions and mitochondria as in A. Note how the bootstrap values are much lower within the bottom branch containing mitochondrial ISP than in the upper branch containing ISP2.

To complement the above analysis of mtDNA-encoded proteins of the *aa_3_*-type oxidase, we next examined the molecular evolution of the “Rieske” iron sulfur subunit (ISP) of the cytochrome *bc_1_* complex. This ubiquitous redox protein is coded by the nuclear DNA and therefore does not suffer from the distortions due to the fast mutation rate of mtDNA-encoded proteins [16], [37], [48]. Its precursor form, once imported into mitochondria, matures within the intermembrane space where its catalytic core resides. After implementing structure-based alignments (Fig. S4 in File S1), we noted diverse insertions that are present in the catalytic core of ISP proteins from different lineages, which we have named CIMit - Conserved Indels vs. Mitochondria (Fig. 7 and Fig. S4 in File S1). CIMit3 is the most prominent of these insertions, lying at the surface of bacterial *bc*_1_ complexes [68], [69] with parallel inserts in the partner protein, cytochrome *b* [68]–[72]. This and other indels (according to the definition in Ref. [73]) seem to carry valuable phylogenetic information, enabling the resolution of relationships that are blurred in phylogenetic trees (cf. Figs. 7C and 8). For instance, only *Tistrella* ISP has no residues corresponding to the CIMit5 insertion among the proteins from Rhodospirillaceae (Fig. 7), while in distance trees these proteins appear to be equally close within a sister sub-branch of their mitochondrial homologues (Fig. 8).

*Methylocystis sp. SC*2 and a few other Rhizobiales have a second, longer ISP (ISP2) that resembles the proteins from acetic acid, β- and γ-proteobacteria, with which it clusters together in distance trees (Figs. 7, 8 and S4 in File S1). Contrary to the latter organisms, ISP2 is not present within the *petABC* operon of the *bc_1_* complex but in isolated gene clusters that have no common flanking genes (not shown). Hence, ISP2 may have arisen from gene duplication as reported for the β proteobacterium, *Rubrivivax gelatinosus*, where the two forms of the proteins are interchangeable in the complex [74]. The duplicates of *Rubrivivax* ISP are closely related to each other, as in the case of the multiple ISP forms of *Roseobacter* and other Rhodobacterales (Table S1 in File S1). However, ISP2 and the in-operon ISP1present in the same Rhizobiales organisms are separated by a deep bifurcation in phylogenetic trees, which resembles that seen in *COX*1 trees (Fg. 3B,C cf. Fig. 8). Hence, ISP2 is an ancestral character of α-proteobacteria equivalent to *COX* operons of type a, consistent with their similar phylogenetic distribution (Fig. 6B). Its origin can be traced to the separation of the αβγ lineages, probably after the earliest proteobacterial ISP had evolved in a distinct path from its paralogues of the *b_6_f* complex present in Planctomycetes and Nitrospirales [75] (Fig. 8B). This ancestral form of ISP was in all likelyhood devoid of the abovementioned insertions as in ISP1of *Rhodopseudomonas palustris* BisA53 or *Nitrobacter hamburgensis*, which lie in the most distant branches of phylogenetic trees (Fig. 8A). Of note, these proteins show the single-residue deletion corresponding to CIMit6, which is shared with the ISP proteins of many α-proteobacteria and their mitochondrial homologues (Figs. 7 and 8).

Importantly, the molecular features of ISP proteins provide crucial information for discriminating between the alternative pathways of mitochondrial bioenergy evolution in Fig. 1B. In particular, bacterial organisms possessing an ISP containing the CIMit3B insert (Figs. 7 and S4 in File S1) can now be excluded from mitochondrial ancestry. This applies not only to Rhodobacterales such as *Roseobacter*, but also to *Rhizobium, Sinorhizobium* and *Mesorhizobium* organisms that have *COX* operon type a-II (Table S2 in File S1).

### 6. Analysis of bacteria without *aa*_3_-type cytochrome c oxidase

The analysis conducted so far has exploited bioenergetic systems that are not always present together in extant bacteria (Table S1 in File S1). For example, *Magnetococcus* has no functional *aa_3_*-type cytochrome *c* oxidase but a complete operon for the *bc_1_* complex and the *cbb_3_*-type oxidase (Table S1 in File S1, cf. Ref. [76]). Phylogenetic analysis has shown that the sequence of *Magnetococcus* ISP is rather similar to that of protists' mitochondria, even if it shows some unique amino acid changes (Figs. 8B and S4 in File S1). *Magnetococcus* lies in a deep branch of the evolutionary tree of α-proteobacteria [76], similarly to *Midichloria*, which also has a *cbb_3_*-type oxidase instead of the *aa_3_*-type oxidase of other Rickettsiales [21]. *Midichloria* has an ISP protein with a unique insertion in the conserved cluster-binding region and also an unusually split version of the catalytic, *COX*1-like subunit of *cbb_3_*-type oxidase [21]. These molecular properties seem to indicate a side-path in the phylogenetic relationships with the mitochondrial lineage (cf. Fig. 1B), a possibility strenghtenend by the analysis of the genomic and protein sequences of *cbb_3_*-type oxidase (data not shown). Hence, the scheme in Fig. 1B is consistent with the overall phylogenetic pattern of both *aa_3_*-type and *cbb_3_*-type terminal oxidases.

## Conclusions

Herein, we have followed novel approaches to reconstruct the possible bioenergetic characters of the bacterial ancestors of mitochondria. Rather than taking into consideration all the information that is now available from bacterial and mitochondrial genomes, we have focused on a few proteins that are crucial for bioenergy production in both bacteria and mitochondria and have multiple variants. The diverse molecular forms and genetic organization of bioenergetic systems have been hardly considered in previous studies of phylogenomics; for instance, none of the papers reviewed in Ref. [9] used proteins of energy metabolism. Conversely, recent studies on bacterial oxidases [27], [64] have not considered the complexity of *COX* operons (Figs. 3 and S3 in File S1). Here we have classified this complexity and exploited its most informative aspects to reconstruct the molecular evolution of individual protein components that are encoded by either mtDNA or nuclear DNA of eukaryotes. By integrating the information thus obtained, we have excluded that several bacterial lineages previously proposed to be related to mitochondria could be in the direct line of mitochondrial ancestry, in particular the endocellular obligate parasites of the Rickettsiales group and the photosynthetic organisms *Rhodobacter* and *Rhodospirillum.* Our work indicates that mitochondrial ancestors retained bioenergetic elements of N metabolism and the *bd-*type ubiqinol oxidase,which have been subsequently lost in different paths of convergent evolution (Fig. 1B).

**Figure 9:**
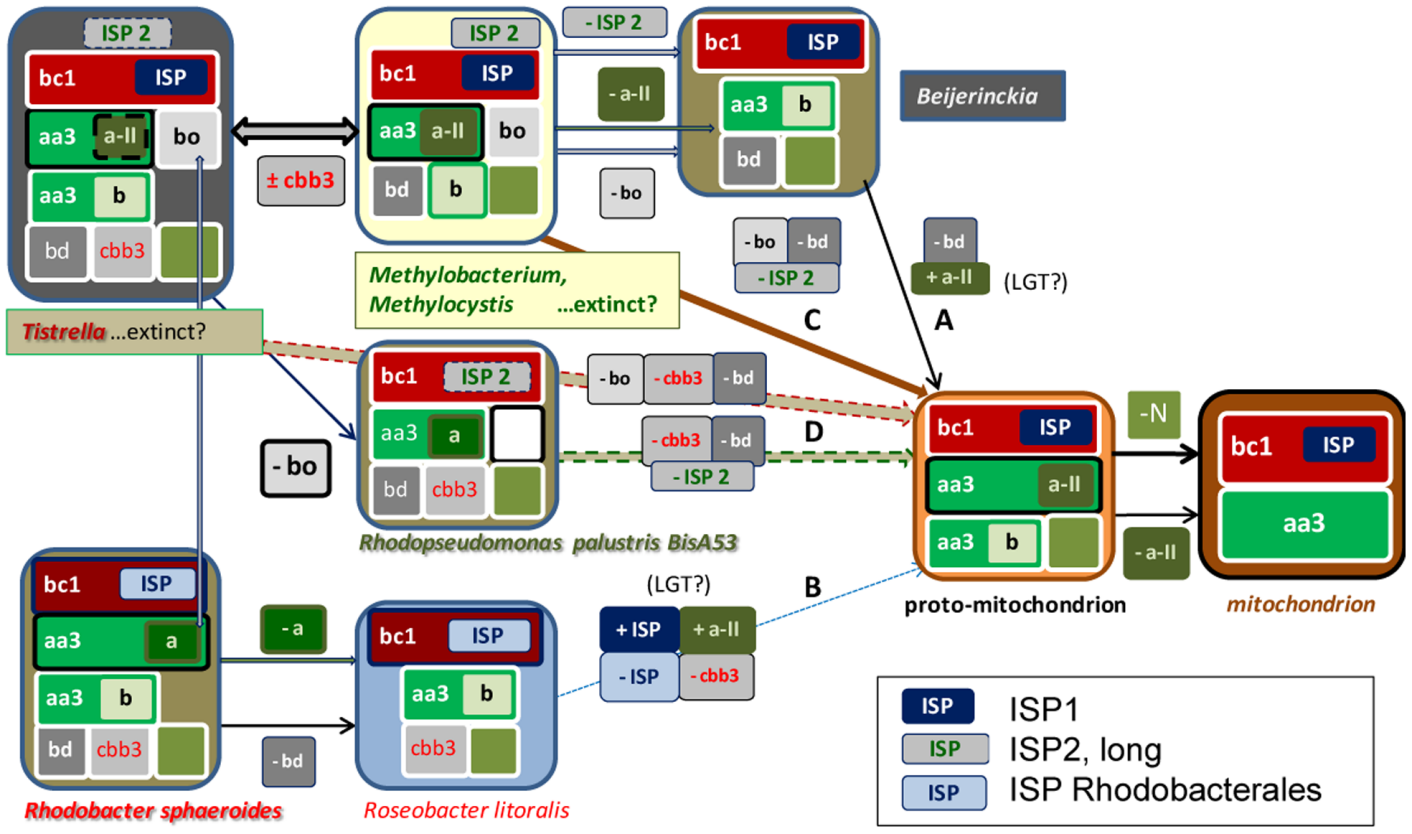
Possible progenitors for the bioenergetic evolution of mitochondria. This diagram is modified from that in Fig. 1B to take into account the deduction that proto-mitochondria probably had two different types of *COX* operons (type a is labelled in dark olive background) and the evidence for multiple ISP forms. ISP2 is represented in a grey box while ISP1 in dark blue. Various steps of differential loss or acquisition via LGT are indicated for the possible pathways of evolution from extant or extinct α-proteobacteria into proto-mitochondria. By considering the complexities arisen from our data, pathway A in Fig. 1B stemming from *Beijerinckia* would require one loss and one acquisition, while pathway B would theoretically imply two losses and two acquisitions. However, we now exclude that this pathway may have contributed to the evolution of mitochondria (see text). Pathway C, sustained by most results presented here, bypasses the *Beijerinckia* subset with the combined loss of two bioenergetic systems and ISP2. Finally, pathway D would require the combined loss of three bioenergetic systems from organisms such as *Tistrella*, but of two systems plus ISP2 for *R. palustris* BisA53, which has already lost *bo*-type oxidase (Table S1 in File S1). The obvious possibility that yet undiscovered, or extinct bacteria may be among the originators of the proto-mitochondrion is considered, as indicated. Eventual loss of photosynthesis is not shown, but it would apply only to *Methylobacterium*, *R. palustris* and *Roseobacter* among the organisms shown. The grey vertical arrow on the left indicates the possible equivalence of *COX* operon type a with dual function (cytochrome *c* and ubiquinol) oxidases in some Rhodobacterales.

In concluding this work, we discuss steps of differential loss also in conjunction with the possible acquisition of systems or proteins via LGT, to provide a complete account of the remaining possibilities for the evolution of mitochondrial bioenergy production (Figure 9). Multiple lines of evidence emerging from our work lead to the conclusion that the subset of bioenergetic systems lacking the *cbb_3_*-type oxidase - typical of methylotrophs and *Gluconacetobacter* (Table S1 in File S1) - probably matches the bioenergetic capacity of the distal ancestors of mitochondria. This evidence includes the maximal diversity of *COX* operons and N metabolism in the abovementioned organisms (Tables S1 and S2 in File S1). The ancestral organisms from which proto-mitochondria emerged in all likelyhood evolved just after the separation of β- and γ-proteobacterial lineages, a concept that is sustained, in particular, by the taxonomic distribution of fused bioenergetic proteins and key elements of N metabolism (Fig. 6). At the whole taxon level, β- and γ-proteobacteria have a much higher frequency of these characters than α-proteobacteria (Fig. 6B). However, some α-proteobacteria show a high frequency of fused proteins and elements of N metabolism (Fig. 6B), namely methylotrophs - encompassing the families of Methylocystaceae, Methylobacteraceae, Beijerinckiaceae and part of Hyphomicrobiaceae, as well as Bradyrhizobiaceae such as *Afipia felis* and *Rhodopseudomonas palustris* BisA53 [77] - several Acetobacteraceae and some Rhodospirillaceae. These organisms also have a wide range of ancestral characters such as type a *COX* operons and ISP2 (Table S2 in File S1 and Fig. 8).

The information just discussed can be integrated with the timeline of bacterial evolution [31], which positions the separation of the β-lineage near the time at which oxygen levels dramatically increased, at least in the photic zone of marine environments and emerged land. The invention of the metabolic pathways of methane, ammonia and nitrite oxidation immediately followed, allowing autothrophic ways of life which are now retained by a few groups of proteobacteria [30]. These bacteria also possess the largest variety of *COX* operons and molecular forms of their catalytic subunits, as the result of multiple events of operon and gene duplication. Some of these duplications are still evident in extanct organisms, as indicated by the doublet of *COX*3 proteins in *COX* operon type a-III (Fig. 3A) and the presence of concatenated *COX* operons in some genomes (Table S2 in File S1). Our reconstruction of the molecular evolution of *COX*3 proteins and their binding strength for oxygen-modulating phospholipids (Fig. 5) seems to recapitulate a progressive adaptation to increasing levels of O_2_, which had to be gauged in terms of decreasing oxygen affinity to maintain maximal efficiency of the oxidase reactions, with minimal damage by radicals and potential suicidal reactions [47], [60], [78]. We have also found multiple forms of other terminal oxidases in methylotrophs and Rhodospirillales, in particular for the *bd*-type ubiquinol oxidase (Table S1 in File S1). The additional forms usually correspond to the Cyanide Insensitive Oxidase (CIO) [79], which has lower affinity for oxygen than classical *bd* oxidases [25].

We believe that the large increase in ambient oxygen that occurred during the evolution of primordial proteobacteria [31] was the driving force for the genomic expansion and diversification of oxygen-reacting enzymes. High levels of O_2_ also led to the wide availability of nitrate and nitrite that can function as alternative terminal acceptors for electron transfer and bioenergy production [22], [32]. This underlines the strong link between oxygen respiration and key elements of N metabolism that we have taken in consideration here. The separation of proto-mitochondria is estimated to have occurred when oxygen levels were still very low in the oceans [10], [24], where most primordial life thrived. It is therefore plausible that the distal progenitors of mitochondria were related to organisms that had experimented with a wide variety of oxygen-reacting systems and thus retained great plasticity in their adaptation to micro-oxic or even anoxic environments, a trait that is partially retained in eukaryotes adapted to anaerobic environments [10]. With this conceptual framework in mind, we can now look back to the initial approach of our work (Fig. 1) and consider the most plausible pathways for mitochondrial evolution (Fig. 9).

Following the separation of the β- and γ-proteobacterial lineages, proto-mitochondia may have branched off along one of the pathways illustrated in Fig. 9. Pathways A and B are the same as in Fig. 1B, with the additional complexities that have emerged from the detailed analysis of COX operons and ISP proteins plus possible acquisitions via LGT. Pathway A, stemming from *Beijerinckia* (we now exclude *Micavibrio* for it lacks key elements of N metabolism, cf. Fig. 2), would require one loss (*bd* oxidase) plus one acquisition (*COX* operon type a-II), while pathways B would theoretically require two losses and two acquisitions of bioenergetic systems. However, our results indicate that mitochondrial evolution is unlikely to have followed pathway B, since the organisms from which it departs do not have key elements of N-metabolism that are present in some eukaryotes (Figs. 2 and 6B) nor a ISP comparable to that of eukaryotes (Figs. 7 and 8). Additional pathway C bypasses the *Beijerinckia* subset with the combined loss of two bioenergetic systems and ISP2, the latter being a facile evolutionary step for only six organisms have retained ISP2 (Figs 7 and 8). This pathway stems from methylotrophic bacteria such as *Methylocysists* and *Methylobacterium*. Indeed, the analysis of three different types of bioenergy-producting systems - cytosolic nitrate assimilation, mitochondria-encoded subunits of cytochrome *c* oxidase and nuclear-encoded ISP subunit of the cytochrome *bc_1_ c*omplex – converges in indicating methylotrophs as the most likely relatives to proto-mitochondria. Moreover, by combining the analysis of nitrate metabolism (Fig. 2) with that of *COX* (Figs. 3–6) and ISP evolution (Figs. 7, 8 and S4 in File S1), only *Tistrella* [48] and *Rhodopseudomonas palustris* [6] remain among all the bacteria that have been previously proposed as possible ancestors of mitochondria (cf. Figs. 1B and Table S1 in File S1). We have thus considered also pathway D, which would require the combined loss of three bioenergetic systems from those possessed by *Tistrella* (Fig. 9). Finally, *Rhodopseudomonas palustris* BisA53 does not have the *bo*-type oxidase as other organisms of the same genus, but possesses a methanol dehydrogenase close to that of methylotrophs (Table 1). However, it still retains a photosynthetic system, the loss of which would add to the other steps required to resemble proto-mitochondria (Fig. 9). The obvious possibility that yet undiscovered, or extinct bacteria may be among the originators of the proto-mitochondrion is also considered in Fig. 9. Yet, these unknown organisms would probably have the subsets of bioenergy systems shown in the top part of the diagram.

Taken all our results together, methylotrophic organisms emerge as the closest living models for mitochondrial ancestors. In perspective, our work provides new means for selecting bacterial organisms that are most suitable for experimentally re-evolving proto-mitochondria with mitochondria-depleted eukaryotic cells.

## Methods

To identify genes and their products with others currently present in National Center for Biotechnology Information (NCBI) resources, we have extensively used the program DELTABLAST, Domain Enhanced Lookup time Accelerated BLAST [80], integrated with hydropathy analysis conducted with in house algorithms [72] or the program WHAT (Web-based Hydropathy, Amphipathicity and Topology http://saier-144-21.ucsd.edu/barwhat.html [81]). Manually refined alignments of bioenergetic proteins were subjected to phylogenetic analysis with maximum likelihood algorithm and 100 bootstrap resamplings, using the program PhyML 3.0 and evolutionary models selected with Prottest3, as described earlier [21]. The results obtained with this rigorous method essentially matched those obtained with the recent options of DELTABLAST (cf. Fig. 8). The genomes of *Asaia platicody* and *Saccharibacter sp.* (EMBL accession: CBLX010000001/27 and CBLY010000001/09, respectively) were recently reported by Chouaia *et al* [22]. See Supporting Information for additional methods and procedures of gene recognition, operon classification (cf. [82]) and sequence analysis of proteins (cf. [41], [52], [83]).

## Supporting Information

File S1 **We enclose File S1 with Supporting Information containing a detailed account of the classification of bacterial COX operons (2 pages), 4 additional Figures and 4 additional Tables. Figure S1, Pathways for the bioenergetic evolution of α bacterial not leading to mitochondria.** The diagram shows the additional subsets of bioenergetic systems that are not shown in Fig. 1B, including those of *Asaia* and *Saccharibacter* (Table S1B in File S1). The asterisk* labels the same subset as in Fig. 1B (main text), but with fewer representative taxa. Underlined organisms are symbionts or pathogens. Each of the six bioenergetic systems presented in Fig. 1 was identified from its catalytic protein subunits and was considered functionally absent when one or more of these subunits were not found in their completeness, as indicated by the profile of their conserved domains (cf. [41]). The functional absence of a given system is represented by an empty square as in Fig. 1B. **Figure S2, Sequence analysis to identify the fusion of** ***COX*** **4 subunit with** ***COX*** **1 proteins. A.** Sequences of recognised or putative *COX*4 were manually aligned to reference proteins having known 3D structure around the first transmembrane helix (TM1, highlighted in grey): subunit IV of *Thermus caa_3_* oxidase (accession: pdb|2YEV [54]) and subunit IV (COX4_pro_2 super family [cl06738]) of *Rhodobacter Sphaeroides aa_3_* oxidase (chain D, accession: pdb|1M57 [53]). *Residues in **bold** have positive scores (≥ 0) in the BLOSUM62 substitution matrix [83], those **yellow-highlighted** are identical with either reference protein, while those highlighted in purple are identical to *Janibacter COX*IV (accession: ZP_00994995) with scores ≥ 5 [83]. The total count of identities is also highlighted in yellow (tot) before the description of the protein on the right. It was used to identify other *COX*4-like proteins such as DUF983 (see Fig. 3A and the section entitled “classification of bacterial *COX* operons” in File S1). The minimal count for deeming a protein as “*COX*4-like” was considered to be 10, but several *COX*1 proteins exhibited larger numbers of identities. The region of ciliate *COX*1 showing similarity with *COX*4 partially overlaps the last transmembrane region (TM12) of aligned *COX*1, which is well conserved among all available *COX*1 sequences from ciliates. However, the *COX*4-like region in bacterial *COX*1 and that of the pathogenic fungus *Zymospetoria* [55] lies outside the conserved domains of other *COX*1 proteins. Azospirillum_bras, *Azospirillum brasilense*; Methylobac_extor, *Methylobacterium extorquens*. **B** - This panel shows the alignment of *COX*4 subunits around the second transmembrane helix (TM2), the structure of which is known only for subunit IV of *Thermus caa_3_* [54] that was used as the reference for aligning bacterial *COX*4 and mtDNA-encoded proteins. In **bold black** are the residues that are identical in the aligned position of at least two *COX*4 sequences, or are positive substitutions [83] across at least three aligned *COX*4 sequences; they are additionally **yellow-highlighted** when identical between at least one bacterial *COX*4 and one mtDNA-encoded protein (cf. A). In **bold dark blue** are the residues that are positive substitutions between bacterial *COX*4 and mtDNA-encoded proteins, while those in **bold light blue** are identical or positive substitutions among the aligned mtDNA-coded proteins. This colour labelling enhances the limited similarity between the sequences shown. **Figure S3, Gene sequence of additional** ***COX*** **operons in diverse bacteria**. The reference gene name for each cluster is indicated on the right of the figure. Symbols identify the same proteins as in Fig. 3A, with the addition of the small gray bar, protein related to nucleotide exchange factor EF-TS. These short proteins were recognised after alignment to the sequence with known 3D structure of Chain A, dimerization domain of Ef-Ts from *Thermus thermophilus* (Accession: pdb|1TFE|A) using a sequence analysis similar to that shown in Fig. S2 in File S1. Hypothetical steps in the evolution of *COX* operons are indicated. **Figure S4, Structure-based alignment of bacterial and mitochondrial “Rieske” ISP**. The protein sequences of various ISP of the *bc_1_* complex were aligned following structures available from various sources matching the alignment gaps or insertions with the most refined 3D data [68]–[71]. The limits of secondary structures (alpha helices, highlighted in green, and beta sheets, highlighted in purple) were deduced from a consensus of the latest coordinates deposited in the NCBI databanks [68]–[71]. Common insertions and deletions (Indels [72]) between mitochondrial and bacterial sequences are consecutively labelled CIMit1-7 (cf. Fig. 7A). The C terminus of some sequences is truncated at the residue indicated by the numeral before the slash. Key residues for the iron-sulfur cluster, including Y165 influencing its redox potential [71], are in **bold**. Note that *Nitrospira, Nitrosomonas*, *Nitrosococcus* and *Methylocystis* are metabolically related by ammonia/methane autothropy. The organisms follow established phylogenetic distance from top to bottom according to the following taxonomic groups and species. **Cyanobacteria**: Synechocystis (*b_6_f* complex), *Synechocystis* sp. PCC 6803, 192 aa; **Nitrospirales**: Nitrospira, *Candidatus Nitrospira defluvii* [73], 183 aa; **ε-proteobacteria**: Epsilon, *Helicobacter pylori*, 167 aa; **Planctomycetes:** Kuenenia_2, *Candidatus Kuenenia stuttgartiensis* (in-operon Kuste3096 [66]), 173 aa; Schlesneria_2, *Schlesneira paludicula* DSM 18645 (accession: ZP_11092182), 189 aa. **γ-proteobacteria**: Nitrosoc, *Nitrosococcus watsonii* C-113, 201 aa; Frateuria, *Frateuria aurantia*, 201 aa; **β-proteobacteria**: Nitrosomonas, *Nitrosomonas europaea* ATCC 19718, 201 aa; Beta, *Neissseria meningitidis* MC58, 193 aa. **α-proteobacteria:** Methylocy_1 &_2, *Methylocystis* sp. SC2 [84], _1 in-operon,176 aa, _2 in isolated gene cluster, 209 aa; Methylob_r, *Methylobacterium radiotolerans* JCM 2831, 189 aa; Nitrobacter, *Nitrobacter hamburgensis* ISP2, 219 aa; Gluc_dia, *Gluconacetobacter diazotrophicus* PAl 5 (in isolated gene cluster), 221 aa; Saccharib, *Saccharibacter* sp. (Chouaia *et al*. [22]), 223 aa; Glu_oxyd, *Gluconobacter oxydans* H24, 218 aa; Beijerinckia, *Beijerinckia indica*, 172 aa; RoseobacterA2, *Roseobacter litoralis petA2* in-operon, 186 aa;Maricaulis_1, *Maricaulis maris* in-operon, 207 aa; Micavibrio, *Micavibrio aeruginosavorus* [25], 185 aa; Magnetococcus, *Magnetococcus marinus* [76], 178 aa; Rickettsia, *Rickettsia felis*, 177 aa. **Mitochondria:** **Acanthamoeba**, *Acanthamoeba castellanii*, 235 aa; S_cerevisiae, *Saccharomyces cerevisiae*, mature 185 aa (3D structure available [85]); Chicken, *Gallus gallus*, mature 192 aa (3D structure available [68]). C-terminal extensions are highlighted in pale blue with some conserved residues in gray. **Table S1, Genomic distribution of bienergetic systems in α-proteobacteria. A.** The genomes of *ca.* 120 α-proteobacterial organisms were studied from the latest version of the genome NCBI database http://www.ncbi.nlm.nih.gov/genome/browse/- accessed on 14 March 2014,verifying also the completeness of genomic data (*). Reconstruction of the various bioenergetic systems (see text) was deduced by combining genomic information with biochemical and microbiological data. The organisms are listed following the left-right sequence in the model of Fig. 1B. Major types of *bd* oxidases are classified as bd-I or CIO [25], [79]. The organisms directly shown in Fig. 1B are yellow highlighted and those proposed to be relatives of mitochondria are shown in italics with pertinent references (including [86], [87]). Underlined organisms are symbionts or pathogens. **B**. This table lists the organisms that have been analysed but are not included in the model of Fig. 1B, also because they are in parallel paths of evolution with respect to the mitochondrial subset of bioenergetic systems. The organisms highlighted in pale yellow are shown in Fig. S1 in File S1, while other annotations are the same as in **A**. Complementary information is in Table S2 in File S1. **Table S2, Diverse gene clusters for** ***aa_3_*** **-type oxidase in α-proteobacteria.** The table lists the diverse types of *COX* operons (Fig. 3A). *COX*1 proteins recognised as ba3-like_Oxidase_I [cd01660] [41] are under the column ba3∧ and correspond to class B [26]. Concatenated operons are framed in blue and connected by a thick line. Incomplete (or ‘dead’ [82]) operons, indicated by the asterisk*, lack one or more of core subunits *ctaC-E* (Fig. 3A). Functional capacity of the oxidase has been deduced also from biochemical studies [88], [89]. **Table S3, Phylogenetic distribution of the main characters of** ***COX*** **gene operons.** We constructed a matrix of 11 independent characters (indicated concisely on top of the columns) that could differentiate the gene sequence of *COX* subunits in the mitochondria of some protists from the gene sequence of bacterial *COX* operons. The cumulative phenetic analysis indicate that *COX* operon type a-II of methylotrophs and *Tistrella* (highlighted) share the largest number of characters with *COX* gene clusters of protist mitochondria (F. Comandatore and C. Bandi, unpublished). **Table S4, Conserved phospholipid binding sites in** ***COX*** **3 proteins.** The alignment in Fig. 4A was enlarged and the residues corresponding to the PL-binding sites and E90 (close to O2 entry in beef *COX*3 [60]) were considered conserved when producing positive substitutions [83] (bold amino acid symbols in white background). Other substitutions are highlighted in pale brown while identities are identified as **yes**. Organisms are abbreviated as in Fig. 4.
(PDF)
