# Supplementary material for: Evolution of Mitochondria Reconstructed from the Energy Metabolism of Living Bacteria
Source: PLoS One. 2014 May 7;9(5):e96566. doi: 10.1371/journal.pone.0096566 (PMC4013037; doi:10.1371/journal.pone.0096566)
Supplement: File S1 — We enclose File S1 with Supporting Information containing a detailed account of the classification of bacterial COX operons (2 pages), 4 additional Figures and 4 additional Tables. Figure S1, Pathways for the bioenergetic evolution of α bacterial not leading to mitochondria. The diagram shows the additional subsets of bioenergetic systems that are not shown in Fig. 1B, including those of Asaia and Saccharibacter (Table S1B in File S1). The asterisk* labels the same subset as in Fig. 1B (main text), but with fewer representative taxa. Underlined organisms are symbionts or pathogens. Each of the six bioenergetic systems presented in Fig. 1 was identified from its catalytic protein subunits and was considered functionally absent when one or more of these subunits were not found in their completeness, as indicated by the profile of their conserved domains (cf. [41]). The functional absence of a given system is represented by an empty square as in Fig. 1B. Figure S2, Sequence analysis to identify the fusion of COX 4 subunit with COX 1 proteins. A. Sequences of recognised or putative COX4 were manually aligned to reference proteins having known 3D structure around the first transmembrane helix (TM1, highlighted in grey): subunit IV of Thermus caa3 oxidase (accession: pdb|2YEV [54]) and subunit IV (COX4_pro_2 super family [cl06738]) of Rhodobacter Sphaeroides aa3 oxidase (chain D, accession: pdb|1M57 [53]). *Residues in bold have positive scores (≥ 0) in the BLOSUM62 substitution matrix [83], those yellow-highlighted are identical with either reference protein, while those highlighted in purple are identical to Janibacter COXIV (accession: ZP_00994995) with scores ≥ 5 [83]. The total count of identities is also highlighted in yellow (tot) before the description of the protein on the right. It was used to identify other COX4-like proteins such as DUF983 (see Fig. 3A and the section entitled “classification of bacterial COX operons” in File S1). The minimal count for deeming [file pone.0096566.s001.pdf]

Supporting Information

We enclose this File S1 of Supporting Information containing a detailed account of the classification of bacterial *COX* operons (2 pages), 4 additional Figures and 4 additional Tables.

Classification of bacterial *COX* operons

The terminal oxidases considered here (Fig. 1) belong to the super-class of heme-copper oxygen reductases, which are classified on the basis of structural-functional features of their common catalytic subunit [26], corresponding to *COX*1 or *ctaD* for the aa_3 cytochrome *c* oxidases. Type A includes the majority of these oxidases, while type B includes the ba_3 oxidases [26,47] that lack *COX*3 and *COX*4 in their core gene sequence. Type C correspond to the cbb_3 oxidases, which also lack *COX*3 and *COX*4. Finally, NOR includes the nitric oxide reductases, which are considered under the system of N metabolism here (Fig. 1). While maintaining the framework of this classification, we introduce a new subdivision of the aa_3 cytochrome *c* oxidases which accounts for the large variety in their genomic sequences organized in operons [46,47,51], herein called '*COX* operons' (Figs. 3 and S4, Table S2). We have now classified them systematically in three groups (or sub-types of the overall category A [26]): **type a**, conserving the core sequence *ctaC-F*; **type b**, with the core sequence intermixed with genes for the assembly of the oxidase cofactors, e.g. *CtaB*; and **a-b transition**, showing features intermediate between type a and b and restricted to β- and γ- proteobacteria. Additionally, the ba_3 oxidases (type B [26]) are maintained as a separated type of *COX* operon (Table S2), even though their gene sequences shows features related to both *COX* operon a-b transition and the operon of cbb_3 cytochrome *c* oxidases. From the composition and positioning of the genes that are prepended [82] (i.e. upstream) and appended [82] (i.e. downstream) to the core sequence, we distinguish four different *COX* operons of type a. Type **a** alone is characterized by *COX*1 fused with *COX*3 and the presence of tri-heme *c* cytochromes (Fig. 3 and data not shown). Type **a-I** is associated with the Act (Alternative Complex Three) gene clusters containing homologues of the nitrite reductase *nrfD* [49,50] (Figs. 3A and S4); similarly to ba_3 oxidases, the combined operon is predominantly present in Rhizobiales and Rhodospirillales (Fig. 6A). Type **a-II** is recognized for the fusion of *COX*4 at the C-terminus of *COX*1 (Fig. S2), the fusion of *COX*2 with a *c*

cytochrome and the frequent association with PQQ-dependent dehydrogenases; its richness in mono- and di-heme *c* cytochromes suggests possible evolutionary relationships with the operon of cbb_3 oxidases. Finally, type **a-III** characteristically contains a doublet of *ctaE*/*COX*3 proteins (Figs. 3A and S4) and is widely distributed among α-proteobacteria (Table S2). Although *COX* operons of type a are present also in β- and γ-proteobacteria (Tables S2-S4), *COX* operon type **b** is present only in α-proteobacteria, showing no fusion among *COX* subunits and more variation in the contiguity of the gene sequence than in the gene sequence itself. Indeed, while *Beijerinckia* or *Tistrella* have a continous gene sequence, Rhodobacterales and other taxa present different fragmentations of the operon in separate gene clusters (Figs. 3A and S4A). Of note, previous phylogenetic studies on aa_3 cytochrome *c* oxidases predominantly used the proteins belonging to *COX* operon type b [6,7,13,47,64].

With regard to the possible evolution of *COX* operons, we hypothesize that an early duplication of *COX*4 might have been crucial in the progressive breaking apart of the operon core sequence and the major structural transition of *COX*3 from the 5-helices to the 7-helices form (Fig. S3), presumably by fusion of a two-helices *COX*4-like protein. This large molecular change has enhanced *COX*3 binding to specific lipids, in particular PG1 and PG2 [60] (see alsoTable S4). The resulting dense lipo-protein packing could modulate the entry of oxygen in the catalytic centre [60], thereby reducing the affinity for the oxygen substrate. This molecular change affecting the affinity for oxygen appears to correspond to the juncture between *COX* operons type a-II lacking a separate *COX*4 subunit and the transition a-b type (Figs. 3 and S3). We surmise that such a junction might have been concurrent with the separation of the β- and γ-lineages from primordial α-proteobacteria (Fig. 3C), an event which occurred around the time when oxygen levels strongly increased on the planet [31].

Supporting Information Figures – and their legend

Figure S1 - Pathways for the bioenergetic evolution of α bacterial not leading to mitochondria. The diagram shows the additional subsets of bioenergetic systems that are not shown in Fig. 1B, including those of *Asaia* and *Saccharibacter* (Table S1B). The asterisk* labels the same subset as in Fig. 1B (main text), but with fewer representative taxa. Underlined organisms are symbionts or pathogens. Each of the six bioenergetic systems presented in Fig. 1 was identified from its catalytic protein subunits and was considered functionally absent when one or more of these subunits were not found in their completeness, as indicated by the profile of their conserved domains (cf. [41]). The functional absence of a given system is represented by an empty square as in Fig. 1B.

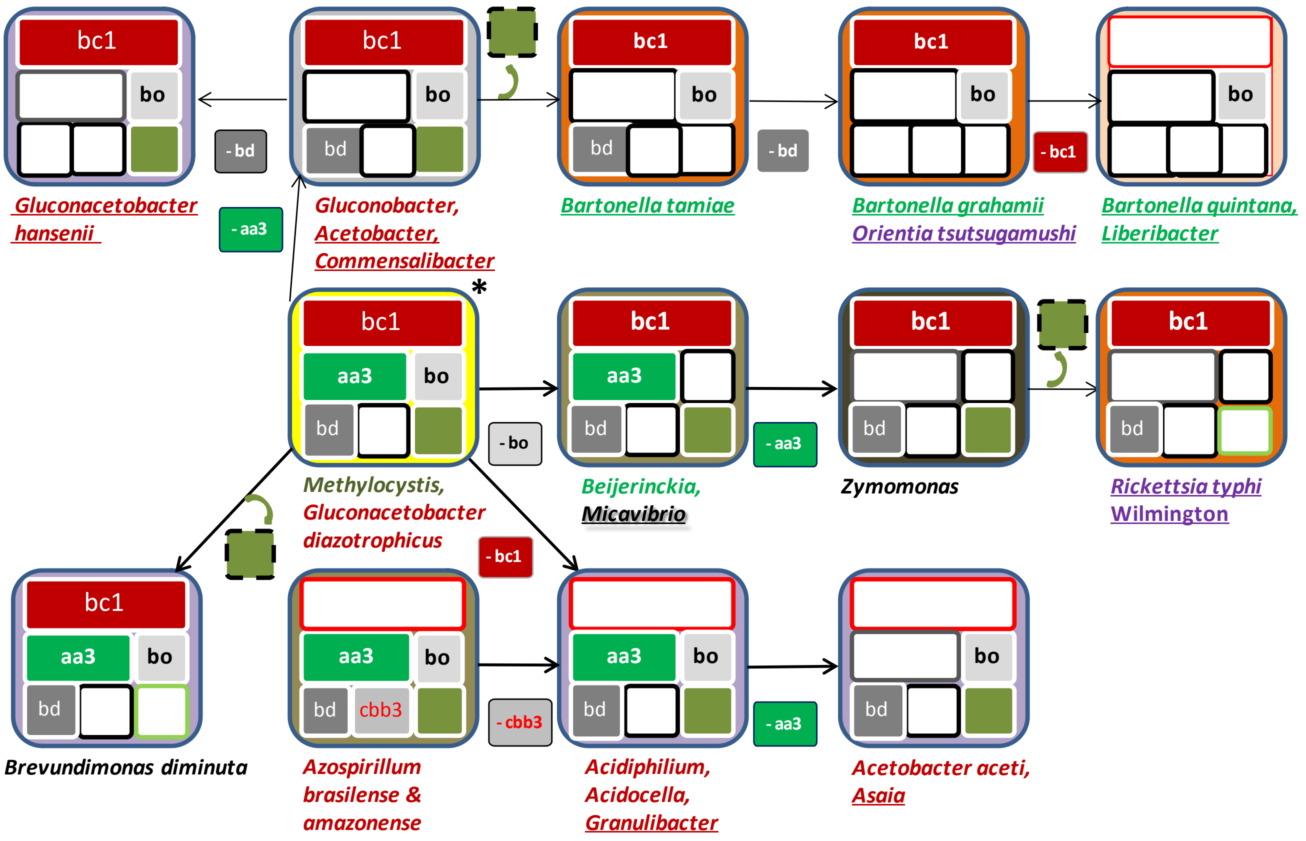

Fig. S2. Sequence analysis to identify the fusion of *COX*4 subunit with *COX*1 proteins.

A. Sequences of recognised or putative *COX*4 were manually aligned to reference proteins having known 3D structure around the first transmembrane helix (TM1, highlighted in grey): subunit IV of *Thermus caa*$_3$ oxidase (accession: pdb|2YEV [54]) and subunit IV (COX4_pro_2 super family [cl06738]) of *Rhodobacter Sphaeroides aa*$_3$ oxidase (chain D, accession: pdb|1M57 [53]). *Residues in **bold** have positive scores (≥ 0) in the BLOSUM62 substitution matrix [83], those **yellow-highlighted** are identical with either reference protein, while those highlighted in purple are identical to *Janibacter COXIV* (accession: ZP_00994995) with scores ≥ 5 [83]. The total count of identities is also highlighted in yellow (tot) before the description of the protein on the right. It was used to identify other *COX*4-like proteins such as DUF983 (see Fig. 3A and the section entitled "classification of bacterial *COX* operons"). The minimal count for deeming a protein as "*COX*4-like" was considered to be 10, but several *COX*1 proteins exhibited larger numbers of identities. The region of ciliate *COX*1 showing similarity with *COX*4 partially overlaps the last transmembrane region (TM12) of aligned *COX*1, which is well conserved among all available COX1 sequences from ciliates. However, the *COX*4-like region in bacterial *COX*1 and that of the pathogenic fungus *Zymospetoria* [55] lies outside the conserved domains of other *COX*1 proteins. Azospirillum_bras, *Azospirillum brasilense*; Methylobac_extor, *Methylobacterium extorquens*. **B.** This panel shows the alignment of *COX*4 subunits around the second transmembrane helix (TM2), the structure of which is known only for subunit IV of *Thermus caa*$_3$ [54] that was used as the reference for aligning bacterial *COX*4 and mtDNA-encoded proteins. In **bold black** are the residues that are identical in the aligned position of at least two *COX*4 sequences, or are positive substitutions [83] across at least three aligned *COX*4 sequences; they are additionally **yellow-highlighted** when identical between at least one bacterial *COX*4 and one mtDNA-encoded protein (cf. A). In **bold dark blue** are the residues that are positive substitutions between bacterial *COX*4 and mtDNA-encoded proteins, while those in **bold light blue** are identical or positive substitutions among the aligned mtDNA-coded proteins. This colour labelling enhances the limited similarity between the sequences shown.

A

```
Accession:                          TM1-----------------------------
pdb|1M57|J     12   HVAGSMDITTQQEKTFAGFVRMVTWAAVVIVAALIFLALANA--- 51                 aa3  subunit IV 3D  Rhodobacter_sphaeroides
pdb|2YEV       2    -VYIALFALGAALVTLFFYLILNP--RVLTTEGETFD-------- 34                 caa3 subunit IV 3D  Thermus

                                                                  ≥0* identical tot                          COX operon type
YP_006592792   10   SVASLMFALLAAELAATFS--FPGWGRSGVAVIAAAMVGIAAFGF  24  14   3    17   Cox 4   Methylocystis SC2         a-I

YP_005030786   582  TIWPLLSAL-AVTVLFIGSI-FTPWALVW-GAVPLAVGLV-GWFW  22  14   8    22   Cox 1   Azospirillum_bras         a-II
WP_009763216   630  SIWPFIAAIATA-ILFIGSM-FTPWALVW-GSVPLALALM-GWFW  22  12   8    20   Cox 1   Microvirga                a-II
YP_006593115   588  SIWPLIAA--AVTITFVGSI-FTPWAVVW-GGALVA-APLIGWFW  21  11   8    19   Cox 1   Methylocystis SC2         a-II
YP_001637594   589  SIWPLVAALAVG-ATFIGSI-FTPYAVLW-GAPPIAAALT-GWFW  25  11   7    18   Cox 1   Methylobac_extor PA1      a-II
YP_006373319   588  SFRPLAAAIFIG-TCFIAAT-FEWWWLAAAG-GIIGIGVILGWLW  17   4   7    11   Cox1/3  Tistrella mobilis         a-II
ZP_00994995    89   SWWPLWLAGSCA-FIFLGLA--VGWWLVALGAALVVPALV-GWVF  23  12  13    25   Cyt_c_ox_IV Janibacter            caa3

YP_001648742   593  VGDVTSRQIYSALIHSMTTVMPTPWNPTLLG-GILQTGNAIVWSA  18   9   3    12   Cox 1   Zymoseptoria tritici   mtDNA
gb|ABI51751    577  QFYLG-FSGMP-RRIHDYPVVFMGWHSMSTTG--HFITLI-GIMF  20   8   5    13   Cox 1   Tetrahymena pigmentosa mtDNA
YP_003734424   632  MFFLG-FSGLP-RRIHDFPAFFLGWHGLATCG--HFLTMI-GVVF  20   7   5    12   Cox 1   Paramecium caudatum    mtDNA
AEV66698       1209 MLWLG-YAGMP-RRVLDYPAAFGGWHSVISAG--HMLNVA-GLMA  21   6   5    11   Cox 1   Oxytricha trifallax    mtDNA
ACX30949       850  MIWLG-YLGMP-RRVLDYPAALGGWHSLVSSAHMI---TVSGILA  21   6   5    11   Cox 1   Moneuplotes minuta     mtDNA

AAA71993       472  HLLGL--AGMP-RRIPDYPDAYLHFNLISSYGSFVTLVSTI-MFF  14   1   2     3   Cox 1   Physarum mtDNA – negative control
                    End of TM12 of mitochondrial COX1
                                                          identities >5* also with COX4 Janibacter
```

B

```
Accession:                        TM2----------------
pdb|2YEV      35   -----------LRFVLFMLLLILLAA-GTVALMLLIGKAHHLL-------------------------    66   Thermus caa3 Subunit IV

YP_006592792  52   --FMDLRQEGVVVRL-FAAAALLWLVILLGLGALDPMTRTLYPTVIAVP--------------------   97   Methylocystis SC2 COX operon a-I
WP_019086563  46   --FMRITRAP-ALAFIFAVGGLFWLSILLALGSLDSFTRTNFPVGDTLSSTAHDPRTPPDTAP-------  105  Gluconacetobacter europaeus  a-I
YP_002961361  60   -LFMRLNRADHLVRL-TAACGLFWLAILFALTLTDTLSRLANT--------------------------  100  Methylobac_extor AM1 & PA1   a-II
WP_009763218  53   ---LSLFGGFLSWRS-YAAAAPTPRTDTTGAGRPHRFMALIGVSIALLFTVIIL-LHGVAGLVFQGCER-  116  Microvirga        COX operon a-II
WP_014239625  65   -----------SWRARQPGKGRAWDSDP-GNRRFVAGL-SAGTAAFFILVILAQALAA---VFLTGCER-  113  Azospirillum_bras COX operon a-II
YP_001769834  54   -VAASLGGSYASWRACQRPPEADATTVPAPDGSRATSLDSPEGGRPHRLIAAIGVLVSLLFAVVIGVHG-  122  Methylobacterium sp. 4-46    a-II
YP_006591292  56   -LFILV-AVLVALRS-QRFRSASPFVAHVSTILAIAALIAGAWTLFPALALPHCA---------------  107  Methylocystis SC2 COX operon a-II

gb|ABI5175   615   -FFLMIFDSHIERRACTSTTLGLPRWYKRISY-YIFKIRYLQHNKSKMNGIP------------------  667  COX1 Tetrahymena pigmentosa mtDNA
AEV66698    1249   -FFIMIFDS---LRQAKAATRNNFGVNRYNTRL-NFYIYEIARLTYVQQKALFLFRIIKSSNLKLNNKNY  1320 COX1 Oxytricha trifallax     mtDNA
```

Fig. S3. Gene sequence of additional *COX* operons in diverse bacteria. The reference gene name for each cluster is indicated on the right of the figure. Symbols identify the same proteins as in Fig. 3A, with the addition of ▯, protein related to nucleotide exchange factor EF-TS. These short proteins were recognised after alignment to the sequence with known 3D structure of Chain A, dimerization domain of Ef-Ts From *Thermus thermophilus* (Accession: pdb|1TFE|A) using a sequence analysis similar to that shown in Fig. S2. Hypothetical steps in the evolution of *COX* operons are indicated.

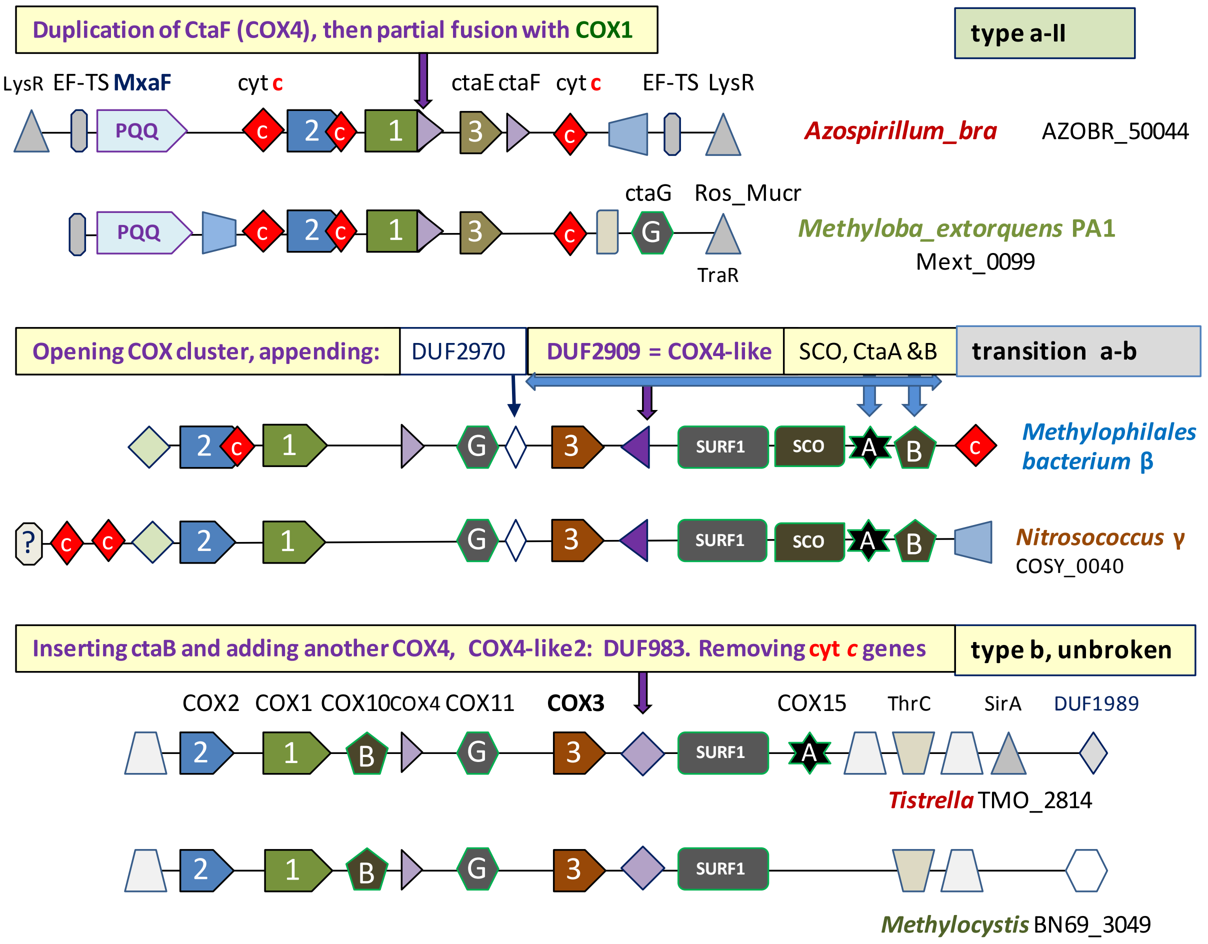

Figure S4. Structure-based alignment of bacterial and mitochondrial “Rieske” ISP.

The protein sequences of various ISP of the bc_1 complex were aligned following structures available from various sources matching the alignment gaps or insertions with the most refined 3D data [68-71]. The limits of secondary structures (alpha helices, highlighted in green, and beta sheets, highlighted in purple) were deduced from a consensus of the latest coordinates deposited in the NCBI databanks [68-71]. Common insertions and deletions (Indels [72]) between mitochondrial and bacterial sequences are consecutively labelled CIMit1-7 (cf.

Fig. 7A). The C terminus of some sequences is truncated at the residue indicated by the numeral before the slash. Key residues for the iron-sulfur cluster, including Y165 influencing its redox potential [71], are in **bold**. Note that *Nitrospira*, *Nitrosomonas*, *Nitrosococcus* and *Methylocystis* are metabolically related by ammonia/methane autothropy. The organisms follow established phylogenetic distance from top to bottom according to the following taxonomic groups and species. **Cyanobacteria:** Synechocystis (*b_6f* complex), *Synechocystis* sp. PCC 6803, 192 aa; **Nitrospirales:** Nitrospira, *Candidatus Nitrospira defluvii* [73], 183 aa ; **ε-proteobacteria**: Epsilon, *Helicobacter pylori*, 167 aa; **Planctomycetes:** Kuenenia_2, *Candidatus Kuenenia stuttgartiensis* (in-operon Kuste3096 [66]), 173 aa ; Schlesneria_2, *Schlesneira paludicula* DSM 18645 (accession: ZP_11092182), 189 aa. **γ-proteobacteria**: Nitrosoc, *Nitrosococcus watsonii* C-113, 201 aa; Frateuria, *Frateuria aurantia*, 201 aa; **β-proteobacteria**: Nitrosomonas, *Nitrosomonas europaea* ATCC 19718, 201 aa; Beta, *Neissseria meningitidis* MC58, 193 aa. **α-proteobacteria:** Methylocy_1 &_2, *Methylocystis* sp. SC2 [84], _1 in-operon,176 aa , _2 in isolated gene cluster, 209 aa; Methylob_r, *Methylobacterium radiotolerans* JCM 2831, 189 aa; Nitrobacter, *Nitrobacter hamburgensis* ISP2, 219 aa; Gluc_dia, *Gluconacetobacter diazotrophicus* PAl 5 (in isolated gene cluster), 221 aa; Saccharib, *Saccharibacter* sp. (Chouaia *et al* [22]), 223 aa; Glu_oxyd, *Gluconobacter oxydans* H24, 218 aa; Beijerinckia, *Beijerinckia indica*, 172 aa; RoseobacterA2, *Roseobacter litoralis petA2* in-operon, 186 aa; Maricaulis_1, *Maricaulis maris* in-operon, 207 aa; Micavibrio, *Micavibrio aeruginosavorus* [25], 185 aa; Magnetococcus, *Magnetococcus marinus* [76], 178 aa; Rickettsia, *Rickettsia felis*, 177 aa. **Mitochondria: Acanthamoeba**, *Acanthamoeba castellanii*, 235 aa; S_cerevisiae, *Saccharomyces cerevisiae*, mature 185 aa (3D structure available [85]); Chicken, *Gallus gallus*, mature 192 aa (3D structure available [68]). C-terminal extensions are highlighted in pale blue with some conserved residues in gray.

Rieske ISP, N-terminal part: UCR TM domain, hinge & beta1-3 plus major TURN

```
Synechocystis   6    LKFRRFIMTQ---ISGSPDVPDLGRRQFMNLL-TFGTITGVAAGALY-PAVKYLIPPSSGGSGGGVTAKDALGNDVKVTEFLASHNAGDRVLAQGLKGDP
Nitrospira      18   VGSRRTFFHW---VTVAAAAVVGVGLAVPLLGSLISPAFMRRR-----RAWVDVGPVDELPAGRPTQLDHVTTVRDGWQEATSHKAV-------------
Epsilon         3    DIQRRDFLGM---SLASVTAIGAIASLVAMKKTWDPLPSVVSAG----FTTIDVANMQEGQFSTVEWRGKPVYILKRSKKEGFNNEKRDFKI--------
Kuenenia_2      21   TISRRNFLTL---AGWGLFFAAIGAYLSQLFGYKGFFYPKVLF--EPSPRFFVGEPKQFPVGSVTTLKSRKIFVVRDAD---------------------
Schlesneria_2   12   TPPRRTFLAA---ISSVVIGGFITLTPLVSGLAFFLDPVLRTR-----TKFKGGDSEGFLFVANLSDLPDDGTPLRFLPDDGTPLRFQMRADKQDAWNVV
Methylocy_2     8    DPLRRDVLYV---ATGACAVVATAAAIWPLIDALNPDDASSVAAGA--PIDVDLGGLEPGGKVVVRWRGLPVAIFRRPDAALEKLKDPVLL----ALADP
Saccharib       22   RPTRRTVLTA---GSVALGGAGACTLAAPFLDSLRRTRASAASTQEETVLDISVQDLEPGAHKIVVWQGMPVAIQRRTPEMIRALEDPALLA---QLLDP
Glu_oxyd        20   GSRRRDVLAT---VTVAMGCAGACAVAYPFLDSLNGTRANHVGESD--ILDVDLSTLKAGQQIVVTWRGWPVFVQRRTPEMLKTLQDPAILQ---KLRDP
Gluc_dia        20   GTGRRDFLTL---VTLAAGGAGAAAFAWPFLDSLRPGDTKSARA----PVDVDVSKLPPGQQITVVWHGSPVFITHRTPESLARLQEAALAA---RLRDP
Nitrosomonas    10   MSGRRRFLLV---ATSVAGAVAGAGVATPFLRSMMPSERAKAAGA---PVEVDISKLEPGMLLTAEWRGKVVWVLKRTPEMLDNLEKLNSQL-----ADP
Beta            6    NNGRRRFLTL---ATCGAGGVAALGVATPFVASFFPSEKAK-AGA---AVEVDVSKIEAGQLLTAEWQGKPIWVLNRTDQQLKDLKGLNGEL---TNSDP
Nitrosoc        7    DQGKRRFLTA---ATTVVGGVGMGFTAVPFVKSMQPSERAQAAGA---PVEVDISNLEPGHIMTVEWRGKPVWILRRTPEELESIEALTEED---ILRDP
Frateuria       6    DGGRRRFLAV---STVSVGGAGLAAAAVPFAESMQPSARARSLAA---PVQVDLAGIAQEQMVTYAWRGQPVFVVHRSARQLALLQKHDDRL-----LDP
Nitrobacter     17   SPTRRDFLYI---ATAAVGTVGVAAAIVPMISQLEPDKATLAAGG---PVELDLSQVQPGQQVSVRWRDHPILVINRSDAALKTLQDPKLLA---QLSDP
Methylob_r      13   DGSRRDFLFL---ATGAGLAVGAGAAVWPLISSMAPDADTIAAGA---PIEVDLTPIQDGQIVNVFWRGKLIFVRKLTAKEVADMKAVPLS----AMIDP
Rickettsia      11   QTTRRDFMVL---TASSVAAVGAVCTLWPLVDSLNPSADVLALS----SIEVDLSNIAVGQTVTVKWQGKPVFITNRTPDKIAEARAVKMS----ELIDP
Maricaulis_1    14   EPSRRDFIYI---AAGGVAAVGGGLVAWPFIDQMNPAADTLALG----SIRVDVSVVPVGSEITVMWRGGPVFIRHRTEEEIAEARAVELD----ALPDE
RoseobacterA2   8    EGTRRDFLYY---ATAGAGAVTAGAAIWPLVNQMNPTADVLALS----SIRVDVSGIEPGTQLTVKWLGKPVFVRRRTQEEIDAARAVDLA----DLPDQ
Magnetococcus   8    DENRRDFLIM---ATGAVGAVGVAGAAWPFIDSWSPSADVLSLA----TTEVSVAAIAEGQMITTMWQGKPVFILHRNAEQIENLARAKTDDKATDLVDP
Methylocy_1     12   EPSRRDILYI---ATGAAAAGAAAGMVWPLIAQMNPDASTLALA----STEVDLSSVPEGQIVTVKWRGKPVFVRHRTAAEIKSAEDVPLS----ALPDP
Beijerinckia    8    APTRRDFLFI---ATGAMGVVAAGSVLWPLIDQMNPDASTLAAA----TTEVDIGNIAEGQIVTVKWRGKPVFVRHRTKKEISDAEAVPVS----ELRDP
Micavibrio      16   GGTRRDFLFL---TAGAFGAVGAGIFSWPFIDSMNPAADTLAMA----STEVDLSPVEVGQSITVTWRGTPVFIRHRTAEEIEAARAVDVS----SLRDP

Acanthamoeba    62   ESGGRAFSYMVLGTAGVGYAAAAKHTVIKFLDSMNPAANVRAMA----NVEVDVSNIAEGTIMTVKWRGKPLFIRHRTAEEIADAESAPLT----DMRDP
S_cerevisiae    50   ADKGRSYAYFMVGAMGLLSSAGAKSTVETFISSMTATADVLAMA----KVEVNLAAIPLGKNVVVKWQGKPVFIRHRTPHEIQEANSVDMS----ALKDP
Chicken         29   SEDRKGFSYLVTATACVATAYAAKNVVTQFISSLSASADVLALS----KIEIKLSDIPEGKNVAFKWRGKPLFVRHRTQAEINQEAEVDVS----KLRDP   120

Features                 RR     1 α turn less in bacteria
Con.Indels                                                  CIMit1                                       CIMit2
```

Rieske ISP, C-terminal extrinsic part: Iron Sulfur cluster domain to C terminus

```
Synechocystis   --------------TYIVVQGDDTIANYGINAVCTHLGCVVPW----------NASENKFMCPCHGSQYNAEGKVVRG-PAPLSLALAHATVTDDDKLVLSTWTETDFRTDEDPWWA192
Nitrospira      -----------------WAVKQPQGDVTVFSPICTHLGCGYRW----------DDAEKKFLCPCHGSSFDVKGDVLGG-PAPRPLDRLPAKVEGGRLLVMYKDFKSGLRDSVEL   183
Epsilon   79    ----------------------GGSVFTAAIQICTHLGCIPTY----------QDEEKGFLCPCHGGRFTSDGVNIAGTPPPRPFDIPPFKI-EGTKITFGEAGAE...   158/167
Kuenenia_2      --------------------VRDADSFKAISVVCTHLGCAVEF----------SKEKNIFECPCHGSKYYRNGVNFAG-PAPRPLDHLQMIL-DNNGKLAVDTSVKVPLETELIV 173
Schlesneria_2   ANQT---------IGTIYLRKMPGNQIIAFNDTCPHLGCKVDY----------QENSKSFLCPCHASAFKLDGERTNKIPPRGLDTLDSKTDTSGRVWVKYQEFQCGVTQKGAVS 189
Methylocy_2     DSEVL-QQP----RYARNWHRSIDPTYAVLVGVCTHLGCIPLYYPTPSGAEPTADWPGGFFCPCHGSKFDLAGRVYKGVPAPYNLPAPPYRMISDRILRIGENPPGE...  199/209
Saccharib       QSRIR-QQP----REAENAHRSLRPDYGVYVTICTHLGCVPDYQNITS-----LPASGGFFCPCHGSQFDGAGRVLRNMPAPYNLPVPPLHFVDDHTIRLGESLLS...   212/223
Glu_oxyd        ESCIF-QQP----KDATNWHRSVSPDIGVMIGICTHLGCVPTFDAPTQ-----AEPAGKYLCPCHGSQFDSAGRAYRNAPAPYNLPVPPVTMISDTHLRIGESKND...   207/218
Gluc_dia        SSGAL-QQP----PYAANWHRSIKPEFGVVVGICTHLGCVPTYSPAPDPATPIANWPGGYACPCHGSKFDLAGRVFTGAPAPYNLPVPPYSMPGPTTIRLGQNPAG...   210/221
Nitrosomonas    DSQRD-QQP----PYAQNHTRSIKPEILVVLGVCTHLGCSPVYRKDIAPADLGSDWLGGFFCPCHGSKFDLAGRVYKSVPAPSNLVVPPYTYLSDNRLLVGSDSKESA       201
Beta            NSDAE-QQP----EYAKNETRSIKPNILVAIGICTHLGCSPTFRPDIAPADLGADWKGGFFCPCHGSKFDLAGRVYKGVPAPTNLVVPPYKYLSDTTILVGED            193
Nitrosoc        QSEVESQQP----PFAENIYRSIKPELLVVIGICTHLGCSPSFRPEVAPEDLGPDWQGGFFCPCHGSRFDLSGRVYDGVPAPTNLEVPPYRFLAENRLRIGESPSEAT       201
Frateuria       DSKVD-QQP----AWARNPHRSQRPAWLVLVGLCTHLGCVPDYVGSLGREPFDPDWPGGFFCPCHKSRYDLAGRVYSGVPAPRNLLVPPYRFLDDGRLEIGIGPDVPADAQG  201
Nitrobacter     QSSEF-QQP----EYAHNWHRSANPEYGVFVGICTHLGCIPLYYPSPNANHPASDWLGGYFCPCHGSKYDLAGRVFSGVPAPYNLPVPPYRFVSDSTIRIGENPSNSNFNF  210/219
Methylob_r      -------------AAFPTRVKSGHDQWLVVYGNCTHLGCVPIGH---------QGNFEGWACPCHGSQFDAVGRVRHG-PAPINLPIPPYAFETDSKIRIGEGGKEAA       189
Rickettsia      -------------ETDEARVKAGHDNWLVTIGICTHLGCVPLAN---------QGEYDGWFCPCHGSQYDSSGRVRRG-PAPLNLAVPPYTFISDKKIRIG             177
RoseobacterA2   LAENANIAPDA-DASDENRSLDESGEWLVMMGVCTHFGCVPLGD---------AGDYDGWFCPCHGSHYDTSGRIRKG-PAPTNLPVPTAVFVDDTTIQLG             186
Maricaulis_1    DARND-NLPAGVPGTDANRVLR--PEFLIVKGNCTHLGCVPLGLAAN------TGDYDGWYCPCHGSHYDTSARIRRG-PAPENLPVPDYYFETETTVKIGLSPDEASN... 200/207
Magnetococcus   -------------QADADRVKK--DEWLIVLAICTHLGCVPQPI--------GTGDFGGFLCPCHGSHYDTSGRIRKG-PAPRNLEVPYYEFKDDETVIIGKAMG         178
Micavibrio      -------------QTDEDRATK--PEWLVIVGICTHLGCVPLGQKKTDP----HGEFGGWFCPCHGSHYDQSGRIRKG-PAPKNMVVPEYTFLSDTLIRIG             185
Methylocy_1     -------------EPDAKRVQK--PEWLVVIGVCTHLGCIPTGK---------EGEYDGWFCPCHGSVYDLSGRIRSG-PAPTNLEVPPYAFIAENKIKIG             176
Beijerinckia    -------------QTDQSRVQK--PEWLVVIGICTHLGCVPIGH---------EGQYEGWFCPCHGSVYDTSGRIRQG-PAPLNLAVPAYRFVSDTKISIG             172

Acanthamoeba    -------------CPDAARRKADKPEWLIVLGVCTHLGCVPLGG---------QGEYGGWFCPCHGSHYDTAGRIRKG-PAPLNLEVPPYVFKDDSKVLVGVDSV         235
S_cerevisiae    -------------QTDADRVKD--PQWLIMLGICTHLGCVPIGE---------AGDFGGWFCPCHGSHYDISGRIRKG-PAPLNEIPAYEF-DGDKVIVG             185
Chicken   121   -------------QHDLDRVKK--PEWVILVGVCTHLGCVPIAN---------SGDFGGYYCPCHGSHYDASGRIRKG-PAPYNLEVPTYQFVGDDLVVVG          196/272

features        Bac Insert                      C+H+GC+P    αβγinsert     G  C  CHGS Y165                          C-terminus
Con.Indels      CIMit  3a  3b         CIMit4              CIMit5                                   CIMit6           CIMit7
```

Supporting Information Tables

These tables contain large datasets that do no fit the recommended format of the text and are therefore presented as pasted images; they are also available as Excel files, if requested.

Table S1 – Genomic distribution of bienergetic systems in α-proteobacteria.

A. The genomes of *ca.* 120 α-proteobacterial organisms were studied from the latest version of the genome NCBI database http://www.ncbi.nlm.nih.gov/genome/browse/ - accessed on 14 March 2014, verifying also the completeness of genomic data (*). Reconstruction of the various bioenergetic systems (see text) was deduced by combining genomic information with biochemical and microbiological data. The organisms are listed following the left-right sequence in the model of Fig. 1B. Two major types of *bd* oxidases are classified as bd-I or CIO [25,79]. The organisms directly shown in Fig. 1B are yellow highlighted and those proposed to be relatives of mitochondria are shown in italics with pertinent references (including [86,87]). Underlined organisms are symbionts or pathogens. **B.** This table lists the organisms that have been analysed but are not included in the model of Fig. 1B, also because they are in parallel paths of evolution with respect to the mitochondrial subset of bioenergetic systems. The organisms highlighted in pale yellow are shown in Fig. 1, while other annotations are the same as in **A**. Complementary information is in Table S2.

A

Table S1a		BIONERGETIC SYSTEMS					NOTES	Genome* complete	Photosynthetic
		1. bc1	4. aa3	2. bd - types	5. cbb3	3. bo	**Nitrogen (N) metabolism (see also Table 1);** NOR, nitric oxide reductase [26];		
Organism/Name - underlined, endocellular/parasite	Order/Group						**CIO, Cyanide-insensitive bd oxidase [79]**		
proposed mitochondrial relative with Reference									
Shown in Fig. 1B									
All 6 bioenergetic systems									
Paracoccus denitrificans PD1222 [17]	Rhodobacteriales	YES	YES	YES	YES	YES	Nitrate and nitrite reductase AND NOR; CIO bd oxidase; *ba3* as bo oxidase	YES	
Agrobacterium tumefacies F2 [86]	Rhizobiales	YES	YES	YES -bd-I	YES	YES	Nitrate and Nitrite reductase, NOR	YES	
Ochrobactrum anthropi ATCC 49188 [6,7]	Rhizobiales	YES	YES	YES -bd-I	YES	YES	Nitrate and Nitrite reductase, NOR	YES	
Bradyrhizobium ORS 375 [20]	Rhizobiales	YES	YES	YES	YES	YES	Nitrate and Nitrite reductase, NOR like oxidase; CIO bd oxidase; **2 ISP forms**	YES	YES
Rhodopseudomonas palustris HaA2 [6,7]	Rhizobiales	YES	YES	YES - 2 types	YES	YES	Nitrate and Nitrite reductase, NOR; CIO bd oxidase; **2 ISP forms**	YES	YES
Methylobacterium nodulans	Rhizobiales	YES	YES	YES	YES	YES	Nitrate and Nitrite reductase ; CIO bd oxidase	YES	
Microvirga sp. WSM3557	Rhizobiales	YES	YES	YES - 2 types	YES	YES	Multiple COX operons; Nitrate and Nitrite reductase ; CIO bd oxidase	YES	
Rhizobium leguminosarum bv. trifolii WSM2304	Rhizobiales	YES	YES	YES -bd-I	YES	YES	Nitrate and nitrite reductase AND NOR	YES	
Mesorhizobium loti MAFF303099	Rhizobiales	YES	YES	YES	YES	YES	Nitrate and Nitrite reductase, NOR; CIO bd oxidase	YES	
Brucella suis 1330	Rhizobiales	YES	YES	YES -bd-I	YES	YES	Nitrate and nitrite reductase AND NOR	YES	
Methylocella silvestris	Rhizobiales	YES	YES	YES	YES	YES	Methane monoxygenase; Nitrate reductase, **assimilatory Nitrite reductase**; CIO bd oxidase	YES	YES
Hyphomicrobium denitrificans ATCC 51888	Rhizobiales	YES	YES	YES	YES	YES	Nitrate and Nitrite reductase AND NOR; CIO bd oxidase	YES	YES
Parvibaculum lavamentivorans DS-1	Rhizobiales	YES	YES	YES	YES	YES	Nitrate and nitrite reductase AND NOR; CIO bd oxidase	YES	
Tistrella mobilis KA081020-065 [48]	Rhodospirillales	YES	YES	YES -2 types	YES	YES	Nitrate and Nitrite reductase; CIO bd oxidase	YES	
Thalassospira profundimaris WP0211	Rhodospirillales	YES	YES	YES	YES	YES	Nitrate and Nitrite reductase; CIO bd oxidase	NO	
Rhodospirillum centenum SW	Rhodospirillales	YES	YES	YES - 3 types	YES	YES	Nitrate reductase periplasmic and Nitrite reductase; CIO bd oxidase; **2 ISP forms**	YES	YES
Caulobacter [18]	Caulobacteriales	YES	YES	YES -bd-I	YES	YES	Nitrate and Nitrite reductase	YES	
Asticcacaulis excentricus	Caulobacteriales	YES	YES	YES -bd-I	YES	YES	Nitrate and Nitrite reductase AND NOR	YES	
Novosphingobium pentaromativorans	Sphingomonadales	YES	YES	YES -bd-I	YES	YES	Nitrate and Nitrite reductase	YES	
minus 1, bd								YES	
Sphingopyxis alaskensis RB2256	Sphingomonadales	YES	YES	NO	YES	YES	**assimilatory Nitrate & Nitrite reductase**	YES	
minus 1, cbb3								YES	
Gluconacetobacter europaeus LMG 18494	Rhodospirillales	YES	YES	YES - 3 types	NO	YES	Nitrate and Nitrite reductase;**2** CIO bd oxidasea; **2 ISP forms**	YES	
Gluconacetobacter diazotrophicus PAl 5	Rhodospirillales	YES	YES	YES	NO	YES	Nitrate and Nitrite reductase; CIO bd oxidase; **2 ISP forms**	YES	
Azospirillum lipoferum 4B	Rhodospirillales	YES	YES	YES	NO	YES	COX not validated as aa3; Nitrite reductase; CIO bd oxidase	YES	
Acetobacteraceae bacterium AT-5844	Rhodospirillales	YES	YES	YES - 2 types	NO	YES	Nitrate and Nitrite reductase; CIO bd oxidase	NO	
Methylocystis SC2	Rhizobiales	YES	YES	YES - 2 bd-I	NO	YES	Multiple COX operons; Methane monooxigenases; Nitrate and Nitrite reductase; **2 ISP forms**	YES	
Methylobacterium radiotolerans JCM 2831	Rhizobiales	YES	YES	YES	NO	YES	Nitrate reductase periplasmic; Nitrite reductase; 3 CIO bd oxidases	YES	YES
Methylobacterium extorquens (4 strains)	Rhizobiales	YES	YES	YES - 2 types	NO	YES	Nitrate reductase periplasmic; Nitrite reductase; 2 or 3 CIO bd oxidase	YES	YES
Methylosinus trichosporium	Rhizobiales	YES	YES	YES -bd-I	NO	YES	Methane monoxigenase; Nitrate and Nitrite reductase	NO	
Nitrobacter hamburgensis X14	Rhizobiales	YES	YES	YES	NO	YES	Nitrite oxidoreductase and **assimilatory reductase**, NOR like oxidase; CIO bd oxidase; **2 ISP forms**	YES	YES
Azorhizobium caulinodans ORS 571	Rhizobiales	YES	YES	YES - 2 types	NO	YES	Nitrite reductase; CIO bd oxidase	YES	
Sinorhizobium meliloti AK83	Rhizobiales	YES	YES	YES	NO	YES	COX not validated as aa3; Nitrate reductase periplasmic, Nitrite reductase	YES	
Phyllobacterium sp. YR531	Rhizobiales	YES	YES	YES -bd-I	NO	YES	Nitrate and Nitrite reductase	YES	
Pelagibacterium halotolerans B2	Rhizobiales	YES	YES	YES -bd-I	NO	YES	Nitrate and Nitrite reductase	YES	
Parvularcula bermudensis HTCC2503 [18]	Parvularculales	YES	YES	YES -bd-I	NO	YES	Nitrate reductase; CIO bd oxidase; also subunit II of a non functional CIO bd oxidase	YES	
minus 1, bo									
Rhodobacter sphaeroides ATCC 17025 [6-8]	Rhodobacteriales	YES	YES	YES	YES	NO	Nitrate and Nitrite reductase AND NOR; CIO bd oxidase; bo present in other strains	YES	YES
Rhodopseudomonas palustris BisA53 [6,7]	Rhizobiales	YES	YES	YES	YES	NO	Nitrate and Nitrite reductase, NOR; **3 ISP forms**; CIO bd oxidase	YES	YES
Xanthobacter autotrophicus	Rhizobiales	YES	YES	YES	YES	NO	Nitrate reductase and **assimilatory Nitrite reductase**; CIO bd oxidase	YES	
Afipia felis	Rhizobiales	YES	YES	YES	YES	NO	Nitrate and Nitrite reductase, NOR like cyt c oxidase; CIO bd oxidase; **2 ISP forms**	NO	
Brucella abortus S19	Rhizobiales	YES	YES	YES -bd-I	YES	NO	Nitrite reductase only	YES	
Caenispirillum salinarum AK4	Rhodospirillales	YES	YES	YES	YES	NO	Nitrate and Nitrite reductase, NOR; 2 CIO bd oxidases; **2 ISP forms**	NO	YES
Sphingobium sp. SYK-6	Sphingomonadales	YES	YES	YES -bd-I	YES	NO	bo oxidase present in other Sphingobium species; assimilatory Nitrate and Nitrite reductase	YES	YES
minus 2, middle									
Maricaulis maris	Rhodobacterales	YES	YES	NO	YES	NO	Nitrate reductase, respiratory; **2 ISP forms**	YES	
Roseobacter litoralis	Rhodobacterales	YES	YES	NO	YES	NO	Nitrate reductase and assimilatory Nitrite reductase; **3 ISP forms**	YES	YES
Magnetospirillum magneticum AMB-1	Rhodospirillales	YES	YES	NO	YES	NO	Nitrate reductase periplasmic, **assimilatory Nitrite reductase; NOR**	YES	
Micavibrio aeruginosavorus ARL-13 [19]	Rhodospirillales?	YES	YES	YES	NO	NO	Ectoparasite of bacteria; Nitrate reductase, respiratory; CIO bd oxidase	YES	
Beijerinckia indica subsp. indica	Rhizobiales	YES	YES	YES	NO	NO	Nitrate reductase and **assimilatory Nitrite reductase** ; CIO bd oxidase	YES	
minus 2, bottom			YES						
Rhodospirillum rubrum [20]	Rhodospirillales	YES	NO	YES -bd-I	YES	NO	Nitrite reductase in various strains of the species; NOR; **2 ISP forms**	YES	YES
Rhodospirillum photometricum DSM 122	Rhodospirillales	YES	NO	YES -bd-I	YES	NO	Nitrite reducase only; **2 ISP forms**	YES	YES
Oligotropha carboxidovorans OM4	Rhizobiales	YES	NO	YES	YES	NO	Nitrate reductase respiratory, Nitrite reductase; CIO bd oxidase	YES	
minus 3, top									
Rickettsia prowazekii Rp22 [4-7,87]	Rickettsiales	YES	YES	YES	NO	NO	**NO N** metabolism; CIO bd oxidase	YES	
Rickettsia felis URRWXCal2 [4-7,88]	Rickettsiales	YES	YES	YES	NO	NO	**NO N** metabolism; CIO bd oxidase	YES	
Wolbachia sp. wRi	Rickettsiales	YES	YES	YES	NO	NO	**NO N** metabolism; CIO bd oxidase	YES	
Wolbachia endosymbiont of Drosophila melanogaster	Rickettsiales	YES	YES	**YES**	NO	NO	**NO N** metabolism; CIO bd oxidase	YES	
Candidatus Odyssella thessalonicensis L13	Rickettsiales	YES	YES	YES -bd-I	NO	NO	**NO N** metabolism	NO	
alpha proteobacterium BAL199	Rickettsiales	YES	YES	YES	NO	NO	**NO;** CIO bd oxidase	NO	
Mitochondrion, fungi & protists	eukaryotes	YES	YES	NO	NO	NO	**Assimilatory Nitrate and Nitrite reductases**	*YES*	
minus 3, bottom									
Magnetococcus marinus MC-1	Magnetoccocales	YES	NO	NO	YES	NO	COX1 similar to NOR, but COX2 and COX3 missing; Nitrate reductase periplasmic	YES	
Candidatus Midichloria mitochondrii IricVA [21]	Rickettsiales	YES	NO	YES	YES	NO	**NO N** metabolism; CIO bd oxidase	YES	
Candidatus Endolissoclinum faulkneri L5	Rhodospirillales	YES	NO	NO	YES	NO	Nitrite reductase	YES	
minus 4, middle									
Anaplasma marginale str. St. Maries	Rickettsiales Anap.	YES	YES	NO	NO	NO	**NO N** metabolism	YES	
Wolbachia endosymbiont of Culex quinquefasciatus NIV	Rickettsiales Anap.	YES	YES	NO	NO	NO	**NO N** metabolism	YES	
Ehrlichia canis str. Jake	Rickettsiales Anap.	YES	YES	NO	NO	NO	**NO N** metabolism	YES	
Neorickettsia risticii str. Illinois	Rickettsiales Anap.	YES	YES	NO	NO	NO	**NO N** metabolism	YES	
Candidatus Pelagibacter ubique HTCC1062 [13,14]	SAR clade	YES	YES	NO	NO	NO	**NO N** metabolism	YES	
Candidatus Puniceispirillum marinum IMCC1322 [13,14]	SAR11 clade	YES	YES	NO	NO	NO	**NO N** metabolism - a sulfte reductase remotely related to assimilatory nitrite reductases	YES	
SAR116 cluster HIMB100 [13]	SAR11 clade	YES	YES	NO	NO	NO	**NO N** metabolism	NO	
Mitochondrion - metazoans	eukaryotes	YES	YES	NO	NO	NO	**NO N** metabolism	*YES*	

B

Table S1b		BIONERGETIC SYSTEMS					Nitrogen (N) metabolism (see also Table 1); NOR, nitric oxide reductase;	Genome*	Photosynthetic
		1. bc1	4. aa3	2. bd -types	5. cbb3	3. bo	CIO, Cyanide-insensitive bd oxidase [79]	complete	
Organism/Name	Order/Group								
shown in Fig. S1									
All 6 bioenergetic systems									
Starkeya novella DSM 506	Rhizobiales	YES	YES	YES	YES	YES	Nitrate and Nitrite reductase; CIO bd oxidase	YES	
Rhodovulum sp. PH10	Rhodobacteriales	YES	YES	YES - 2 types	YES	YES	Nitrite reductase; 2 CIO bd oxidases; **3 ISP forms**	NO	YES
Azospirillum sp. B510	Rhodospirillales	YES	YES	YES -bd-I	YES	YES	Nitrate reductase periplasmic and Nitrite reductase	YES	
Roseomonas cervicalis ATCC 49957	Rhodospirillales	YES	YES	YES - 3 types	YES	YES	NOR; 2 CIO bd oxidases	NO	
Thalassospira xiamenensis M-5 = DSM 17429	Rhodospirillales	YES	YES	YES	YES	YES	Nitrate reductase periplasmic, **assimilatory Nitrite reductase;** CIO bd oxidase	NO	
minus 1									
Azospirillum amazonense Y2	Rhodospirillales	NO	YES	YES - 2 types	YES	YES	No ISP in bc1; Nitrate reductase periplasmic, Nitrite reductase; CIO bd oxidase	NO	
Azospirillum brasilense Sp245	Rhodospirillales	NO	YES	YES	YES	YES	NO cytochrome b nor c1; Nitrate reductase periplasmic, Nitrite reductase; CIO bd oxidase	YES	
Roseomonas sp. B5	Rhodospirillales	YES	YES	YES - 2 types	NO	YES	Nitrate and Nitrite reductase; CIO bd oxidase	NO	
Magnetospirillum gryphiswaldense MSR-1 v2	Rhodospirillales	YES	YES	YES	YES	NO?	COX possibily working also as ubiquinol oxidase; Nitrate and nitrite reductase; CIO bd oxidase	YES	
Oceanibaculum indicum P24	Rhodospirillales	YES	YES	YES -bd-I	YES	NO	Nitrate reductase	NO	
Gluconacetobacter oboediens 174Bp2	Rhodospirillales	YES	NO	YES - 3 types	YES	YES	**assimilatory Nitrite reductase**; 2 CIO bd oxidases; **2 ISP forms**	NO	
Fulvimarina pelagi	Rhizobiales	YES	YES	YES	NO	YES	Nitrate and Nitrite reductase; CIO bd oxidase	NO	YES
Polymorphum gilvum SL003B-26A1	Polymorphum	YES	YES	YES - 2 types	YES	NO	Nitrate and Nitrite reductase; CIO bd oxidase	YES	
minus 2									
Acetobacter pomorum	Rhodospirillales	YES	NO	YES - 2 types	NO	YES	Nitrate reductase; CIO bd oxidase	NO	
Acetobacter pasteurianus IFO 3283-01-42C (9 strains)	Rhodospirillales	YES	NO	YES - 2 types	NO	YES	Nitrate reductase and **assimilatory Nitrite reductase** ; CIO bd oxidase	YES	
Gluconacetobacter xylinus NBRC 3288	Rhodospirillales	YES	NO	YES	NO	YES	Nitrate and Nitrite reductase ; CIO bd oxidase; **2 ISP forms**	YES	
Gluconobacter oxydans 621H	Rhodospirillales	YES	NO	YES	NO	YES	COX not functional; **assimilatory Nitrite reductase**; CIO bd oxidase	YES	
Gluconobacter morbifer	Rhodospirillales	YES	NO	YES	NO	YES	COX not functional; Nitrite reductase; 2 CIO bd oxidases	NO	
Saccharibacter sp.	Rhodospirillales	YES	NO	YES	NO	YES	COX not functional; Nitrite reductase; CIO bd oxidase	YES	
Saccharibacter floricola	Rhodospirillales	YES	NO	YES -bd-I	NO	YES	COX not functional; Nitrite reductase		
Commensalibacter intestini A911	Rhodospirillales	YES	NO	YES -bd-I	NO	YES	COX not functional; Nitrite reductase; CIO bd oxidase; **2 ISP forms**	NO	
Acidiphilium cryptum JF-5	Rhodospirillales	NO	YES	YES 2 types	NO	YES	cytochome b deranged and NO ISP; **assimilatory Nitrate and Nitrite reductase**; CIO bd oxidase	YES	YES
Acidocella	Rhodospirillales	NO	YES	YES 2 types	NO	YES	cytochome b & ISP deranged; respiratory Nitrate reductase & **assimilatory Nitrite reductase**; CIO bd oxidase	YES	
Granulibacter bethesdensis CGDNIH1	Rhodospirillales	NO	YES	YES	NO	YES	**assimilatory Nitrate and Nitrite reductase**; CIO bd oxidase	YES	
Brevundimonas diminuta	Caulobacteriales	YES	YES	YES -bd-I	NO	YES	**NO N** metabolism	NO	
Phaeospirillum molischianum	Rhodospirillales	YES	NO	YES -bd-I	YES	NO	Nitrite reducase only; **2 ISP forms**	YES	YES
Nitratireductor indicus C115	Rhizobiales	YES	NO	YES -bd-I	YES	NO	**assimilatory Nitrate reductase,** Nitrite reductase	NO	
Rhodomicrobium vannielii ATCC 17100	Rhizobiales	YES	NO	YES	YES	NO	Nitrite reductase; CIO bd oxidase	YES	YES
Magnetospirillum magnetotacticum	Rhodospirillales	YES	YES	NO	YES	NO?	COX possibily working also as ubiquinol oxidase; Nitrate and nitrite reductase	NO	
minus 3									
Acetobacter aceti	Rhodospirillales	NO	NO	YES	NO	YES	COX 1 short; Nitrite reductase; 2 CIO bd oxidases, 1 partial	NO	
Asaia platicody	Rhodospirillales	NO	NO	YES -bd-I	NO	YES	COX not functional; **assimilatory Nitrite reductase**	NO	
Asaia sp. SF2.1	Rhodospirillales	NO	NO	YES	NO	YES	nitrite and nitrate reductase; CIO bd oxidase		
Gluconacetobacter hansenii ATCC 23769	Rhodospirillales	YES	NO	NO	NO	YES	Nitrite reductase	YES	
Zymomonas mobilis subsp. mobilis ZM4	Sphingomonadales	YES	NO	YES	NO	NO	Nitrite/sulfite reductase only; CIO bd oxidase	YES	
Bartonella tamiae (2 strains)	Rhizobiales	YES	NO	YES	NO	YES	**NO N metabolism -** fragments of proteins related to Nitrite reductase; CIO bd oxidase	NO	
minus 4									
Orientia tsutsugamushi str. Boryong	Rickettsiales	YES	YES	NO	NO	NO	**NO N** metabolism	YES	
Rickettsia typhi str. Wilmington	Rickettsiales	YES	NO	YES	NO	NO	**NO N** metabolism; CIO bd oxidase	YES	
Bartonella grahamii as4aup	Rhizobiales	YES	NO	NO	NO	YES	**NO N** metabolism	YES	
minus 5									
Bartonella quintana (2 strains)	Rhizobiales	NO	NO	NO	NO	YES	**NO N** metabolism	YES	
Bartonella clarridgeiae 73	Rhizobiales	NO	NO	NO	NO	YES	**NO N** metabolism	YES	
Bartonella bacilliformis KC583	Rhizobiales	NO	NO	NO	NO	YES	**NO N** metabolism	YES	
Bartonella henselae	Rhizobiales	NO	NO	NO	NO	YES	**NO N** metabolism	YES	
Candidatus Liberibacter asiaticus str. psy62	Rhizobiales	NO	NO	NO	NO	YES	**NO N** metabolism	YES	

Table S2. Diverse gene clusters for aa_3 -type oxidase in α-proteobacteria. The table lists the diverse types of *COX* operons (Fig. 3A). *COX*1 proteins recognised as ba3-like_Oxidase_I [cd01660] [41] are under the column ba3^ and correspond to class B [26]. Concatenated operons are framed in blue and connected by a thick line. Incomplete (or 'dead' [82]) operons, indicated by the asterisk*, lack one or more of core subunits *ctaC-E* (Fig. 3A). Functional capacity of the oxidase has been deduced also from biochemical studies [88,89].

	COX operon *aa3- type* cytochrome *c* oxidase							
**Group and organism**	**type a**	**a-I**	**a-II**	**a-III**	**ba3^**	**type b**	**a-b transition**	**NOTES**
**Beta proteobacteria**								
Nitrosomonas europea					yes*		yes	
Burkholderia xenovorans LB400	yes		yes		YES		yes	
Variovorax paradoxus S110	yes		yes				yes	
Methylophilales bacterium HTCC2181							yes	
**Alpha proteobacteria**								
**Rhodospirillales**								
Gluconacetobacter europaeus LMG 18494		yes				yes		
Gluconacetobacter diazotrophicus PAl 5	yes	yes				yes		
Acetobacteraceae bacterium AT-5844	yes		yes	yes		yes		
Roseomonas sp. B-5	yes	yes				yes		incomplete genome
Roseomonas cervicalis	yes					yes		incomplete genome
Tistrella mobilis		yes	yes			yes		
Caenispirillum salinarum		yes			YES	yes		
Magnetospirillum gryphiswaldense MSR-1	yes - dead*				yes*	yes		
Magnetospirillum magnetotacticum					yes*	yes - 1 dead* & 1 complete		
Magnetospirillum magneticum AMB-1					yes*	yes - 1 dead* & 1 complete		
Magnetospirillum sp. SO-1					yes*	yes - 1 dead* & 1 complete		
Azospirillum brasilense	yes		yes			yes		
Azospirillum lipoferum	yes					yes - 2		
Rhodospirillum centenum SW	yes					yes		
Azospirillum amazonense						yes		
**Rickettsiales**								
Anaplasma phagocytophilum HZ						yes - 2 (1 split, 1 dead*)		
Ehrlichia ruminantium str. Welgevonden						yes - 2 (1 split, 1 dead*)		
**Rhizobiales**								
Nitrobacter winogradsky	yes					yes - 2		
Nitrobacter hamburgensis		yes				yes		
Bradyrhizobium sp. S23321					YES	yes		plus other 3 *Bradyrhizobium* species
Bradyrhizobium sp. WSM471	yes - dead*		yes	yes	YES	yes		
Bradyrhizobium sp. ORS 375		yes	yes - 2			yes		
Bradyrhizobium japonicum USDA 6			yes			yes		
Bradyrhizobium sp. ORS 285			yes			yes		plus other 3 *Bradyrhizobium* species
Bradyrhizobium sp. ORS 278					YES	yes		
Rhodopseudomonas palustris BisA53 (& DX-1)	yes					yes		plus other 3 *Rhodopseudomonas* strain
Afipia felis	yes - dead*					yes		incomplete genome
Afipia birgiae	yes - dead*			yes		yes		incomplete genome
Methylocystis sp. SC2	yes - 2	yes	yes	yes		yes		
Methylocystis parvus	yes	yes		yes		yes		incomplete genome
Methylosinus trichosporium	yes					yes		
Microvirga	yes		yes	yes		yes		
Methylobacterium extorquens PA1		yes	yes			yes		
Methylobacterium extorquens DM4		yes			YES	yes		plus other 3 *Methylobacterium* species
Methylobacterium sp. 4-46	yes		yes			yes		
Methylobacterium nodulans		yes	yes	yes	YES	yes		
Methylobacterium radiotolerans	yes - 2					yes		
Methylobacterium populi		yes				yes		plus another *Methylobacterium* species
Chelatococcus sp. GW1	yes - dead*		yes	yes		yes		incomplete genome
Methylocella silvestris			yes			yes		
Methyloferula stellata			yes		yes*	yes		incomplete genome
Beijerinckia indica						yes		
Starkeya novella	yes			yes		yes		
Pseudaminobacter salicylatoxidans	yes - dead*					yes		
Aurantimonas manganoxydans	yes		yes			yes		
Pelagibacterium halotolerans B2	yes					yes		
Parvibaculum lavamentivorans DS-1	yes					yes		
Sinorhizobium meliloti 1021	yes			yes		yes		
Chelativorans sp. BNC1	yes		yes	yes		yes		
Sinorhizobium medicae WSM419	yes		yes			yes		
Mesorhizobium opportunistum WSM2075		yes	yes - 2			yes		plus other 3 *Mesorhizobium* species
Rhizobium leguminosarum bv. trifolii WSM1325		yes	yes	yes		yes		plus other 5 *Rhizobium* species
Rhizobium etli CFN 42/mimosae			yes		YES	yes		plus other 4 *Rhizobium* species
Mesorhizobium ciceri biovar biserrulae WSM1271			yes			yes		
**Rhodobacterales**						yes		
Rhodobacter sphaeroides 2.4.1	yes					yes		
Oceaniovalibus guishaninsula		yes				yes		
Rhodovulum sp. PH10		yes				yes		
Roseobacter litoralis						yes		
**Other groups**								
Brevundimonas subvibrioides	yes					yes		
Phenylobacterium zucineum HLK1	yes					yes		
Sphingobium sp. SYK-6	yes					yes		
Sphingobium chlorophenolicum L-1			yes, dead*	yes		yes		plus other 4 *Sphingobium* species
Sphingobium japonicum UT26S				yes		yes		
Novosphingobium pentaromativorans				yes		yes		
Novosphingobium sp. PP1Y				yes		yes		
Candidatus Pelagibacter sp. IMCC9063				yes		yes		plus several marine metagenome organisms
Micavibrio						yes		
	concatenated				^Type B, [26]	*incomplete operon, likely non-functional		

Table S3 – Phylogenetic distribution of the main characters of *COX* gene operons.

We constructed a matrix of 11 independent characters (indicated concisely on top of the columns) that could differentiate the gene sequence of *COX* subunits in the mitochondria of some protists from the gene sequence of bacterial *COX* operons (Table S2). The cumulative phenetic analysis indicate that *COX* operon type a-II of methylotrophs and *Tistrella* (highlighted) share the largest number of characters with *COX* gene clusters of protist mitochondria (F. Comandatore and C. Bandi, unpublished).

	CHARACTERS-->	1	2	3	4	5	6	7	8	9	10	11	total
			CtaC-F core									in operon	characters
organism and COX1 length	COX operon type	COX2COX1	COX3 in	E90 in COX3	Cox3 fused with COX1	COX3 with 7TM	COX11COX3	COX4 NOT in	COX4 fused COX1	COX2 fused	no ctaB	ABC transporter	vs. mitos
gamma proteobacteria													
Methylococcus capsulatus, 541 aa	transition a-b	1	1	0	0	0	1	0	0	1	1	0	*5*
Methylobacter marinus, 642 aa	a	1	1	0	1	0	0	0	1	1	1	0	*6*
beta proteobacteria													
Methylophilales bacterium , 531 aa	transition a-b	1	0	1	0	0	0	0	0	1	0	0	*3*
Variovorax paradoxus, 662 aa	a-II	1	1	1	0	0	0	0	1	1	1	1	*6*
alpha proteobacteria													
Acidiphilium cryptum	transition a-b	1	0	1	0	1	0	0	0	0	0	0	*3*
Rhodopseudomonas palustris HaA2,	a	1	1	1	0	1	0	0	0	0	0	0	*4*
Methylocystis sp. SC2, 636 aa	a-II	1	1	1	0	0	0	0	1	1	1	0	*7*
Mesorhizobium opportunisticum, 658 aa	a-II	1	1	1	0	0	0	0	1	1	1	1	*7*
Microvirga sp. WSM3558, 670 aa	a-II	1	1	1	0	0	0	0	1	1	1	0	*7*
Methylocella sylvestris, 650 aa	a-II	1	1	1	0	0	0	0	1	1	1	1	*7*
Methylobacterium extorquens PA1, 638 aa	a-II	1	1	1	0	0	1	1	1	1	1	1	*9*
Rhizobium leguminosarum bv. Trifolii, 637 aa	a-II	1	1	1	0	0	1	1	1	1	1	0	*8*
Tistrella mobilis, 839 aa	a-II	1	1	1	1	0	0	0	1	1	1	0	*7*
Azospirillum brasilense, 645 aa	a-II	1	1	1	0	0	0	0	1	1	1	0	*6*
Beijerinckia indica subsp. Indica, 555 aa	b	1	0	1	0	1	1	1	0	0	0	0	*5*
Micavibrio aeruginovorous, 559 AA	b	1	0	1	0	1	1	1	0	0	0	0	*5*
Magnetospirillum magneticum AMB-1	b	1	0	1	0	1	1	1	0	0	0	0	*5*
Maricaulis maris	b	1	0	1	0	1	1	1	0	0	0	0	*5*
Rhoseobacter litoralis	b	0	0	1	0	1	1	0	0	0	0	0	*3*
Wolbachia wRi	b	1	0	1	0	1	0	1	0	0	0	0	*4*
Rickettsia felis	b	0	0	1	0	1	0	1	0	0	0	0	*3*
eukaryotes (mitochondria)													
Dictyostelium		1	0	1	0	1	0	1	0	1	1	0	*6*
Jakobida		1	0	1	0	1	1	1	0	0	1	1	*7*
Tetrahymena		1	0	1	0	0	0	0	1	0	1	0	*4*
Oxythricha		1	1	1	1	1	0	0	1	1	1	0	*8*
	CHARACTERS	1	4	6	3	5	7	8	8	9		11	

Table S4 – Conserved phospholipid binding sites in COX3 proteins.

The alignment in Fig. 4A was enlarged and the residues corresponding to the PL-binding sites and E90 (close to O2 entry in beef COX3 [60]) were considered conserved when producing positive substitutions [83] (bold amino acid symbols in white background). Other substitutions are highlighted in pale brown while identities are identified as yes. Organisms are abbreviated as in Fig. 4.

in phylogenetic/phenetic order					PL binding sites				(beef numbering)														
				O2	PG1				PG1 &PG2 PG2								PE2					PE1	PL binding
Organism and protein	protein	Accession	length	E90	W57	W58	E64	F86	H71	T66	F214	R221	F/V233	G234	Y241	H231	Y181	Y182	I185	F198	F203	G82 & M83	total*
			(aa)																				*bold = identities
Methylocystis sp. SC2	COX1-3	YP_006591291	827	L	yes	yes	N	G	L	P	C	A	T	S	L	P	yes	A	gap	A	A	0	3
Rhodo_palu_BisA53	COX1-3	YP_782773	841		yes	P	G	A	G	R	Y	gap	S	A	M	I	T	M	L	G	yes	0	2 & 2
Methylocystis sp. SC2	COX3a	YP_006592539	236	V	M	E	yes	V	yes	gap	A	K	T	R	yes	L	W	R	gap	Y	V	0	3 & 3
Variovorax_par EPS	COX3b	WP_018905232	234	D	gap	gap	gap	yes	Y	gap	yes	K	S	I	yes	N	F	S	gap	yes	yes	0	5 & 4
Methylocystis sp. SC2	COX3	YP_006592793	213	yes	gap	gap	gap	I	yes	G	L	K	N	S	yes	R	W	R	A	yes	L	0	3 & 3
Roseomonas sp. B5	COX3	WP_019459297	217	yes	gap	gap	A	yes	yes	E	V	Q	M	P	yes	Q	yes	H	gap	Y	L	1	5 & 4
Gluconacetobacter_europ	COX3	WP_019086564	210	yes	gap	gap	yes	yes	yes	P	L	yes	P	A	yes	R	W	H	A	Y	L	2	7 & 4
Variovorax_par S110	COX3	YP_002942615	201	yes	gap	gap	gap	L	yes	gap	D	L	V	D	yes	R	F	A	W	W	L	1	3 & 4
Methylocystis sp. SC2	COX3	YP_006593114	242	yes	gap	gap	gap	yes	Y	gap	D	L	S	D	yes	R	F	A	W	W	L	1	3 & 4
Azospirillum brasilense	COX3	YP_005030787	202	yes	gap	gap	gap	yes	yes	gap	D	L	Q	D	yes	R	F	T	W	W	yes	1	5 & 3
Nitrosococcus oceani	COX3	YP_345015	288	yes	yes	F	yes	yes	gap	E	M	yes	yes (F)	A	yes	yes	yes	G	D	Y	yes	2	11 & 3
Methylophi_bac	COX3	WP_008107990	299	yes	yes	F	yes	yes	K	R	M	yes	yes (F)	A	yes	D	yes	M	N	Y	yes	2	10 & 3
Thermus thermopilus	COX1-3	YP_143578	791	yes	yes	A	yes	yes	yes	S	V	L	gap	T	yes	yes	F	yes	H	W	yes	2	10 & 3
mitochondria																							
Plasmodium yoelii	COX3	XP_729225	263	yes	F	V	yes	T	gap	L	yes	D	yes (V)	V	yes	S	L	G	gap	H	T	2	6 & 2
Monoeuplotes minuta	COX1-3	ACX30949	1203	S	gap	F	L	I	gap	yes	Y	L	I	T	V	F	F	D	L		yes	1	4 & 5
Oxytricha trifallax	COX1-3	AEV66698	1331	S	gap	F	Y	gap	F	I	Y	yes	Y	N	yes	W	F	D	yes	yes	N	1	5 & 6
alpha, sister group of mitos																							
Beijerinckia indica	COX3	YP_001831864	287	yes	yes	yes	yes	yes	yes	E	yes	yes	yes	yes	yes	Q	F	A	D	yes	yes	2	14 & 3
Caenispirillum	COX3	WP_009539949	267	yes	yes	yes	yes	yes	yes	M	yes	yes	S	I	W	R	yes	G	A	Y	yes	2	11 & 3
Ricket_prow_Rp22	COX3	YP_005998599	278	yes	yes	yes	yes	yes	yes	I	yes	yes	L	yes	yes	G	yes	yes	E	Y	yes	2	14 & 1
Wolbachia_Dro_sim_	COX3	YP_007886075	275	yes	yes	yes	yes	yes	yes	I	yes	yes	L	C	yes	D	yes	yes	F	Y	yes	1	11 & 1
Paracoccus	COX3	YP_918077	273	yes	yes	yes	yes	yes	yes	E	yes	yes	yes	yes	yes	Q	yes	S	L	Y	yes	2	14 & 4
Roseobacter	COX3	YP_004690241	273	yes	yes	yes	yes	yes	yes	K	yes	yes	yes	yes	yes	K	yes	S	L	yes	yes	2	15 & 2
Tistrella	COX3	YP_006372231	269	yes	yes	yes	yes	yes	yes	Q	yes	yes	I	yes	yes	K	yes	S	T	Y	yes	2	13 & 2
Acidiphilium	COX3	YP_001235282	283	yes	yes	yes	yes	yes	yes	R	yes	yes	yes	yes	yes	R	yes	T	P	yes	yes	2	15 & 1
sister group2: mitochondria																							
Dictyostelium	COX3	NP_050071	435	yes	yes	yes	D	yes	yes	L	T	V	Q	I	yes	N	yes	yes	gap	yes	yes	2	11 & 1
Acanthamoeba	COX3	NP_042532	329	yes	yes	yes	yes	yes	yes	yes	yes	Q	yes (F)	yes	yes	yes	yes	G	gap	H	L	1	13 & 1
Andalucia	COX3	YP_007890482	267	yes	yes	yes	yes	yes	yes	yes	yes	yes	yes	yes	yes	yes	yes	G	gap	H	yes	1	15 & 1
Bos taurus (beef)	COX3	AAZ16929	260	yes	yes	yes	yes	yes	yes	yes	yes	yes	yes	yes	yes	yes	yes	yes	yes	yes	yes	2	19
Organism and protein	protein	operon	aa	E90	W57	W58	E64	F86	H71	T66	F214	R221	F/V233	G234	Y241	H231	Y181	Y182	I185	F198	F203	G82 & M83	total*
